# A global analysis of matches and mismatches between human genetic and linguistic histories

**DOI:** 10.1073/pnas.2122084119

**Published:** 2022-11-21

**Authors:** Chiara Barbieri, Damián E. Blasi, Epifanía Arango-Isaza, Alexandros G. Sotiropoulos, Harald Hammarström, Søren Wichmann, Simon J. Greenhill, Russell D. Gray, Robert Forkel, Balthasar Bickel, Kentaro K. Shimizu

**Affiliations:** ^a^Department of Evolutionary Biology and Environmental Studies, University of Zurich, Zurich 8057, Switzerland;; ^b^Center for the Interdisciplinary Study of Language Evolution, University of Zurich, Zurich 8050, Switzerland;; ^c^Department of Linguistic and Cultural Evolution, Max Planck Institute for Evolutionary Anthropology, Leipzig 04103, Germany;; ^d^Department of Human Evolutionary Biology, Harvard University, Cambridge, MA 02134;; ^e^Human Relations Area Files, Yale University, New Haven, CT 06511-1225;; ^f^Department of Plant and Microbial Biology, University of Zurich, Zurich 8008, Switzerland;; ^g^Department of Linguistics and Philology, University of Uppsala, Uppsala 75126, Sweden;; ^h^Cluster of Excellence ROOTS, Kiel University, Kiel 24118, Germany;; ^i^School of Biological Sciences, University of Auckland, Auckland 1010, New Zealand;; ^j^Department of Comparative Language Science, University of Zurich, Zurich 8050, Switzerland;; ^k^Kihara Institute for Biological Research, Yokohama City University, 244-0813, Yokohama, Japan

**Keywords:** cultural evolution, population genetics, languages, molecular anthropology

## Abstract

Human history is written in both our genes and our languages. The extent to which our biological and linguistic histories are congruent has been the subject of considerable debate, with clear examples of both matches and mismatches. To disentangle the patterns of demographic and cultural transmission, we need a global systematic assessment of matches and mismatches. Here, we assemble a genomic database (GeLaTo, or Genes and Languages Together) specifically curated to investigate genetic and linguistic diversity worldwide. We find that most populations in GeLaTo that speak languages of the same language family (i.e., that descend from the same ancestor language) are also genetically highly similar. However, we also identify nearly 20% mismatches in populations genetically close to linguistically unrelated groups. These mismatches, which occur within the time depth of known linguistic relatedness up to about 10,000 y, are scattered around the world, suggesting that they are a regular outcome in human history. Most mismatches result from populations shifting to the language of a neighboring population that is genetically different because of independent demographic histories. In line with the regularity of such shifts, we find that only half of the language families in GeLaTo are genetically more cohesive than expected under spatial autocorrelations. Moreover, the genetic and linguistic divergence times of population pairs match only rarely, with Indo-European standing out as the family with most matches in our sample. Together, our database and findings pave the way for systematically disentangling demographic and cultural history and for quantifying processes of shifts in language and social identities on a global scale.

## Significance

There has been considerable debate about the extent to which our biological and linguistic histories match to each other, supported by examples of both matches and mismatches. We introduce a genomic database (GeLaTo, or Genes and Languages Together) to quantify matches and mismatches worldwide. While in most populations genetic and linguistic relations match, mismatches occur regularly as a result of language shift, and several language families follow diversification patterns different from that of the genomes. These findings reveal features of population contact in human history that were previously inaccessible to observation. Our database opens avenues for disentangling demographic and linguistic history and for comparing biological and linguistic modes of evolution.

There are numerous conceptual parallels between the processes of genetic and linguistic evolution (1) (here referred to for simplicity as “genes and languages”). In his book *On the Origin of Species*, Darwin went a step further and boldly proposed that the parallels were more than just conceptual. Famously, he claimed that “a perfect pedigree of mankind…would afford the best classification of the various languages now spoken throughout the world” (2, p. 422). The pioneering work of Cavalli-Sforza and Sokal in the 1980s appeared to provide substantial support for Darwin’s claim. The critical evidence for this claim was that a global phylogeny of human populations showed some broad matches with a global language tree (3–5). Genetic and linguistic differentiation processes also appeared to mirror each other on a continental scale in Europe (6, 7). Matches of this kind can result from local codiffusion processes and can be amplified by large-scale population expansions. According to the farming/language dispersal hypothesis, migrations fueled by the shift toward agriculture and animal husbandry in the Holocene have given rise to some of the largest language families identifiable today (8, 9). Notable examples of major language family spreads accompanied by substantial demographic expansions include the Bantu migration in sub-Saharan Africa and the Austronesian peopling of the Pacific. In both cases, genetics and phylolinguistic inference support a broad match of genetic and linguistic histories (10, 11).

In line with this research tradition, research on gene–language associations has tended to emphasize matches between genes and languages, and disregarded mismatches as an exception to the norm. However, regional case studies have repeatedly identified instances where languages and genes clearly do not match (12–15). Mismatches arise if a population adopts another language without (or with only minimal) genetic admixture, or if a population assimilates genetically with a neighboring one without changing its language. For example, Hungarian speakers in central Europe have little or no genetic trace associated with the Siberian origin of their language (16), and Damara speakers in southern Africa have no genetic ties to their linguistically related Nama neighbors (17). While populations necessarily retain the genetic makeup of their ancestors, they can shift to other languages at any time, because speakers can learn new languages throughout their lifespan. Some authors have taken a more extreme position by arguing that language shift has been so pervasive in shaping contemporary linguistic diversity that an association between genes and languages is the exception rather than the rule (18).

However, the claims that either matches or mismatches are the norm are premature. Rather than more cherry-picked examples, what is needed is a systematic assessment of matches and mismatches on a global scale. To accomplish this task, we introduce a global database of gene–language associations: GeLaTo (or Genes and Languages Together), a large, high-resolution genomic resource designed for multidisciplinary research on human cultural and linguistic diversity. We use GeLaTo to address the following questions: How frequent are mismatches between genes and languages? Which scenarios can shape match and mismatch profiles? How genetically cohesive are language families? Within language families, do linguistic and genetic histories reflect the same temporal processes?

## Results

### The GeLaTo Dataset: Coverage, Language Family Distribution, and Population Profiles.

GeLaTo provides genetic and linguistic information for more than 4,000 individuals representing 397 genetic populations speaking 295 languages. Individuals have been genotyped with the Human Origins SNP chip (Affymetrix), which includes ∼500,000 single nucleotide polymorphisms (SNPs) selected to be variable in populations from all the continents; the design of this chip makes it suitable for global scale genetic comparisons. Genetic populations are assigned to a geographic location based on where the samples were collected and to their main spoken language on the basis of linguistic and anthropological information (*Materials and Methods*). Languages are, in turn, assigned to their language families based on a state-of-the-art classification (19). Language families consist of groups of languages that are shown to have derived from a single ancestor language (e.g., English, Italian, Hindi, and many others derive from the single Indo-European proto-language) based on lexical data (*Materials and Methods*). As genetic information, we use pairwise genetic distances estimated via the Weir and Cockerham *F_ST_* (20).

We first quantified the extent to which genetic neighbors speak languages of the same family. This global-scale investigation is subject to constraints of genetic sample coverage, regional structure, and data availability across language families (*SI Appendix*). In response, we first assessed whether populations have enough neighbors from different language families so genetic and linguistic relations could be directly compared. Within a radius of 500 km, more than half of all populations in GeLaTo have a neighboring population in a different language family, and within a radius of 1,000 km, this proportion grows to ∼84%.

Across the whole dataset, we find that for most populations their closest genetic neighbor belongs to the same language family. However, a nonnegligible proportion (18%) is closest to a linguistically unrelated language (Dataset S1). This suggests that mismatches are a regular outcome of language history and not just rare outliers.

### Language and Gene Mismatches at a Local Scale.

**Fig. 1.**
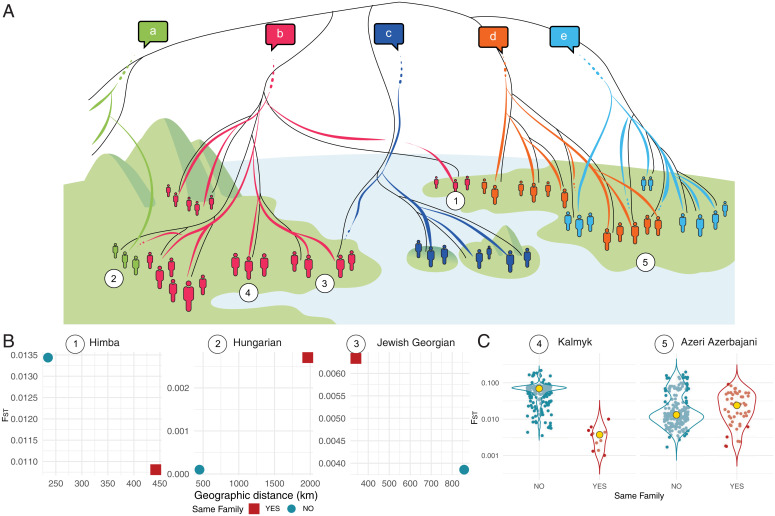
Overview of linguistic and genetic similarity. (*A*) Schematic illustration of possible scenarios of matches and mismatches in the transmission of genes and linguistic traits. Genetic (demographic) history is represented by solid black lines that differentiate groups of people (represented by human shapes). Linguistic history is represented by colored lines, differentiating five language families (*a*–*e*). The linguistic histories sometimes move in parallel with the demographic history and sometimes not. Numbers correspond to the different cases distinguished in *B* and *C*: 1. linguistic and genetic (matching) enclave; 2. linguistic mismatch (linguistic enclave); 3. genetic mismatch (genetic enclave); 4. population with genetic distances aligned with their linguistic relatives (matching profile); 5. population with genetic distances misaligned to their linguistic relatives (mismatching profile). (*B*) Examples of a heuristic associated with the three enclave cases shown in *A*. For each target population, we display the two smallest *F_ST_* distances, respectively, to a population from the same family and a population from a different language family, together with their geographic distance. Himba (Atlantic-Congo family) fulfills the criteria of a matching enclave; Hungarian (Uralic family) fulfills the criteria of a linguistic enclave; Jewish Georgian (Kartvelian family) fulfills the criteria of a genetic enclave. (*C*) Examples of aligned and misaligned cases shown in *A*. For each population, the *F_ST_* distribution within speakers of the same language family is compared with the *F_ST_* distribution between the speakers of other language families. The yellow dot indicates the median. Kalmyk (Mongolic-Khitan) is aligned (i.e., is genetically closer) to speakers of the same language family; Azeri Azerbaijani (Turkic family) is misaligned to speakers of the same language family. *F_ST_* distances are displayed on a logarithmically transformed scale.

We developed two heuristic strategies to identify the different scenarios of matches and mismatches proposed in Fig. 1*A* and Table 1. The first heuristic analysis compares the genetic and linguistic relations that populations have with their closest neighbors and identifies various types of what we call “enclaves.” The signature of enclaves is that they are surrounded by populations that are linguistically and/or genetically different from them, but that they remain similar in those dimensions to other, geographically distant populations (*SI Appendix*). Linguistic and genetic enclaves (or “matching enclaves”) have closer genetic and linguistic relatives in a distant region of the world (see case 1 in Fig. 1*A*); mismatching linguistic enclaves have linguistic relatives in a different region of the world but differ from them genetically (see case 2 in Fig. 1*A*). Mismatching genetic enclaves have closer genetic relatives in a different region of the world but differ from them linguistically (see case 3 in Fig. 1*A*). To identify these cases, we consider each population that belongs to a language family represented in GeLaTo by more than two populations. We determine the closest *F_ST_* for speakers of the same vs. those of a different language family and the relative geographic distance.

**Table 1.** Scenarios associated with matches and mismatches

Scenario	Change in location	Change in language	Change in genetic profile	Reference in Fig. 1*A*
Linguistic and genetic enclave	✓			1
Linguistic enclave	✓		✓	2
Genetic enclave	✓	✓		3
Match, genetically aligned				4
Mismatch, genetically misaligned		✓	✓	5

A total of 52 of these cases correspond to matching enclaves (i.e., they remain similar genetically and linguistically to their geographically distant relatives). 27 turn out to be genetic enclaves, and only one case is identified as a linguistic enclave. Examples of each of these cases are illustrated in Fig. 1*B* (*SI Appendix*, Table S1). This strict heuristic can be calculated only for 20% (*n* = 79 of 397) of all populations in GeLaTo that are not directly related (genetically and/or linguistically) to their neighbors. Moreover, the enclave scenario, as we propose it, does not take into account moderate amounts of gene flow that populations in contact might undergo.

In response to these limitations, we adopt a second heuristic that targets not individual enclaves but language families at a broader scale. We compare the distribution of *F_ST_* distances within and between language families, restricted to the geographic span of the corresponding language family. If the transmission of genes and languages is mostly vertical, we expect the *F_ST_* within families to be overall smaller than the *F_ST_* between them and their neighbors. These populations are defined as genetically aligned with their linguistic relatives (see case 4 in Fig. 1*A*). By contrast, gene flow across linguistic boundaries can lead to some degree of overlap between the two distributions, yielding genetically misaligned populations (see case 5 in Fig. 1*A*). To quantify these two cases, we compute the difference between the median of *F_ST_* between and within language families and associated 95% CIs. This reveals a gradient between populations that are largely aligned and populations that are largely misaligned (*SI Appendix*, Figs. S7 and S8). Examples of aligned and misaligned populations are shown in Fig. 1*C*. The proportion of misaligned populations (having a median *F_ST_* within family larger than the median *F_ST_* between families) is 20%, a proportion that is roughly robust after qualitative screening and consideration of potentially false positives (*SI Appendix*, *SI Text* and Dataset S1) and after evaluating the effects of downsampling within families (*SI Appendix*, Fig. S11).

Finally, we reviewed candidate misaligned populations against available genetic and historical literature, confirming cases of language shift and suggesting new mismatch cases (*SI Appendix*, Figs. S7–S9). Hungarians are possibly one of the most studied cases of mismatch. They are genetically similar to their Indo-European speaking neighbors (*SI Appendix*, Fig. S9) but maintain a separate linguistic identity as a member of the Uralic family (21–23). The Hungarian population preserved the language brought by the Magyars, who conquered the Carpathian Basin in the ninth century CE (21–23), while becoming genetically assimilated to their Indo-European–speaking neighbors through time (*SI Appendix*, Fig. S9). In our dataset, they are the only case of a linguistic enclave (Fig. 1*B*). The Maltese, who are the only Afro-Asiatic speakers in Europe (24), represent a case of potential mismatch not addressed by the genetic literature. The majority of the Maltese speak an Afro-Asiatic language with lexical influences from Italian and English, and they are the only population from the Afro-Asiatic language family in misalignment (Fig. 1*C* and *SI Appendix*, Fig. S7). Their genetic profile can be described as a mix of ancestries from throughout the Mediterranean basin: genetically close to Eastern Sicilian, they share genetic relatedness also with Indo-European speakers from the Balkans and geographically distant Turkish and Middle East Afro-Asiatic speakers (*SI Appendix*, Fig. S9). Finally, previously described cases of language shift are less visible with the larger set of comparisons and higher genetic resolution that is available in GeLaTo. This is the case of the Armenians in the Caucasus. Mitochondrial genetic profiles from Armenian (Indo-European speakers) and Azerbaijan (Turkic speakers) have been described as closer to neighboring Caucasian populations (linguistically unrelated) than to speakers of their respective language families (14). In our dataset, the Azerbaijan-speaking population is indeed misaligned with other Turkic speakers, confirming the proposed mismatch. By contrast, Armenians show an *F_ST_* distribution aligned with that of other Indo-European speakers of Anatolia (*SI Appendix*, Figs. S7–S9), challenging the idea that they shifted their linguistic affiliation.

Overall, our heuristics for identifying enclaves and misaligned populations confirm the regularity of gene–language mismatches across different language families and regions (*SI Appendix*, Fig. S10). It remains unclear, however, whether these mismatches are exceptions to the norm within their language families (as in case *b* in Fig. 1*A*), or whether language families are genetically diverse overall (as in cases *d* and *e* in Fig. 1*A*). To address this question, we next compare genetic profiles at the level of language families.

### Genetic Cohesiveness within Language Families.

To compare the genetic profiles of major language families, we factor in the geographic distance between population pairs, since both languages and genes show spatial autocorrelation effects (5, 7, 25). Language transmission and differentiation might lead to spatial patterns through a number of cultural processes (26). In genetics, the effect of spatial autocorrelation is described by the influential ecological model of isolation by distance (IBD) (27), which predicts a correlation between geographic and genetic distances. This effect has been confirmed in most species, including humans (28, 29).

**Fig. 2.**
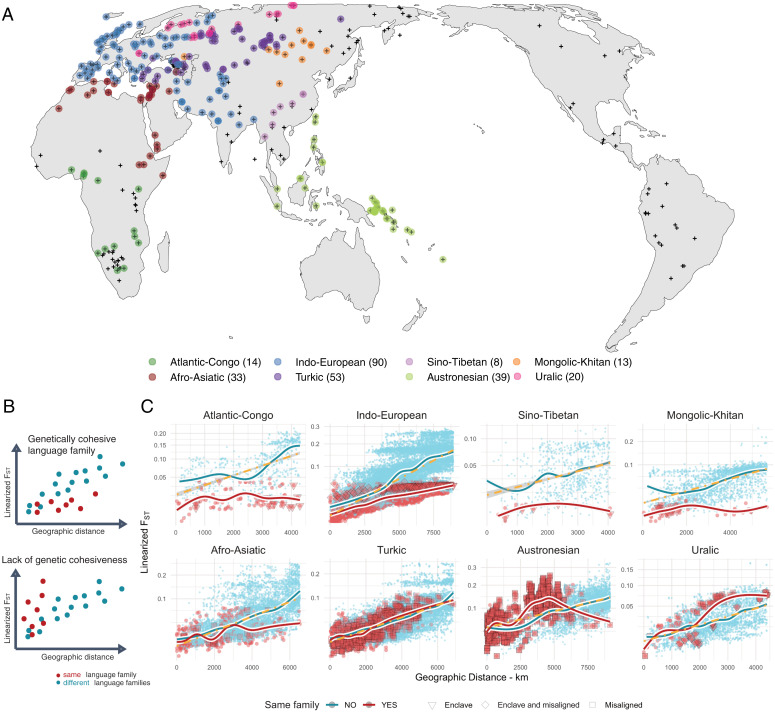
Language family comparisons. (*A*) Approximate location of all the population genetic samples (little black crosses). Target language families are color coded with a solid circle. In the legend, the numbers in parenthesis correspond to the number of population samples for each language family. (*B*) Schematic representation of language family profiles, which are genetically cohesive over geographic distance (match) or which are not genetically cohesive (mismatch). (*C*) Correspondence between genetic distances and geographic distances for eight major language families. In the *Top Row*, language families are mostly genetically cohesive; in the *Bottom Row*, language families show an ambiguous profile. Small blue circles: between-family pairs; large red circles: within-family pairs. Smooth regressions summarize the between- and within-family trends. Different symbols in the same-family comparisons correspond to pairs with populations identified as mismatches, with the heuristics illustrated in Fig. 1 (enclaves and/or misalignments). Yellow dashed line: IBD linear regression between geographic and genetic distances.

Based on this model, we compared *F_ST_* distances within and between families for eight language families that are best represented in GeLaTo (Fig. 2*A*) and correlated them with the respective geographic distance (Fig. 2*C*, yellow line indicating the overall IBD linear correlation). If parallel vertical transmission is dominant, genetic distances between the speakers of the same language family should be smaller than the genetic distance between speakers from different language families, along the same geographic range (Fig. 2*B*). A majority of low within-family *F_ST_* distances over geographic distances supports a tendency for genetic cohesiveness in Atlantic-Congo, Indo-European, Mongolic-Khitan, and Sino-Tibetan. Within-family and between-family distributions roughly overlap for Afro-Asiatic and Turkic, while Uralic and Austronesian are not genetically cohesive, with larger within-family genetic distances over short geographic distances. These patterns do not depend on individual populations or pairs of comparison (as assessed by jackknife and residual analyses; *SI Appendix*, Fig. S12), even when they represent misalignments or mismatches (Fig. 1), when they stand out in the overall distribution of alignments or geographic locations (*SI Appendix*, Fig. S7), or when sample size and geographic dispersion differ substantially between families (Fig. 2*A*). However, families differ in the amount of misalignment and mismatched cases (represented by different shapes in Fig. 2*C*), which show genetic distances systematically larger than what is predicted by the IBD model, even in those families that are, in general, cohesive. Genetic cohesiveness of the language families is therefore better described as a gradient from small to large proportions of mismatches.

### Genetic and Linguistic Similarities over Time.

Genetic cohesiveness within a family is a potential indicator of gene–language association, but it might result from demographic and cultural events taking place at different times (30). We therefore explore the time frame of genetic relatedness within language families and compare it with its reconstructed linguistic time depths. Under the hypothesis of perfect parallel vertical transmission of genes and languages, time-depth estimates are expected to coincide. In particular, the earliest common population ancestor in a given language family should roughly be dated near the root of the language family.

Linguistic time-depth estimates are reconstructed and calibrated on the basis of diverse types of archeological, historical, and linguistic evidence (31). Extant language families vary greatly in their putative ages, ranging from cases that diverged in the last two millennia, such as Quechua, Turkic, and Tungusic, to cases such as Afro-Asiatic, which has been (tentatively) linked by some authors to a pre-Holocene time frame (32) (see *SI Appendix*, *SI Text* for further discussion). Dating methods in linguistics are controversial. For this reason, we use two kinds of linguistic time depths: one combining archaeological, historical, and linguistic quantitative comparisons (*Materials and Methods*), and one calculated using a recently introduced generalized Bayesian dating method (33). Genetic time-depth estimates are derived from the genetic *F_ST_* adjusted for effective population size (*N_e_*) and are calculated over those population pairs in GeLaTo that have a common ancestor at the root level of the linguistic phylogeny. We do not assume a systematic bias in overestimating or underestimating the genetic and linguistic times: for linguistic data, we operate within time depths that can be known or inferred. For genetic data, our *N_e_* calculations are approximate, but we filter for populations with stable population size, even if in the long-term different human groups experienced either expansions or decline.

**Fig. 3.**
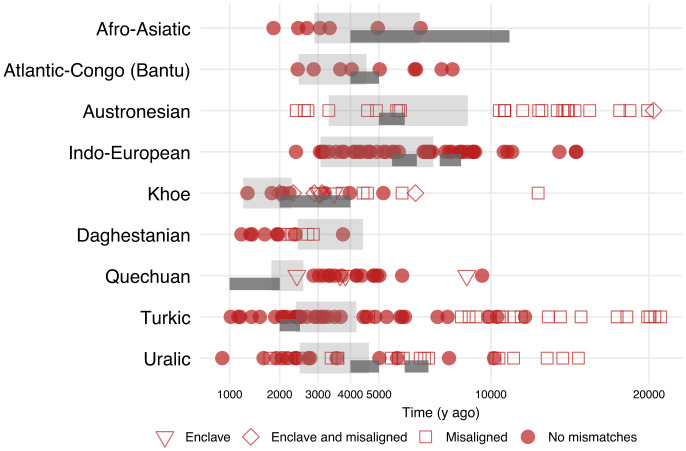
Pairwise divergence time within families or major subgroups. Each point corresponds to the genetic divergent times of population pairs which share a most recent common ancestor at the root level of the language family. Solid circles exclude populations flagged as mismatches. Other symbols indicate pairs which include one type of mismatch (enclave and/or misalignment), following the same conventions as in Fig. 2. Two methods to reconstruct the divergence time of each language family are shown. Light gray blocks correspond to the 95% credible intervals of divergence time reconstructed by generalized Bayesian dating (33). Darker lines below the gray blocks show proposed divergence times from archaeological and historical reconstructions, with indicative time boundaries. Note that such reconstructions are not available for all language families, and, in some cases, two historical reconstructions have been suggested for the same family (see *Materials and Methods* and *SI Appendix*, *SI Text* for references).

No family supports a particularly close match of divergence times (Fig. 3). In some families (namely, Afro-Asiatic, Daghestanian, and Uralic), genetic divergence times tend to be similar to or younger than linguistic time estimates. In all other families, there are mismatches on both sides, with a noticeable trend for genetic divergence times much older than the linguistic time estimates, which are robust with the different estimates employed.

These ancient divergent events suggest prehistorical mismatch events. Different explanations can contribute to this pattern. First, the discrepancy could be due to the lack of power in reconstructing deep genetic and linguistic estimates, which might be associated with larger uncertainty, especially for the genetic time split (*Materials and Methods*). Second, populations with divergent genetic histories can end up speaking linguistically related languages because of language shift (as in the case of genetic enclaves); in particular, admixture with divergent genetic ancestries can contribute to push the genetic reconstruction estimates back in time.

To explore gene–language timescale mismatches in detail, we analyze three high-resolution linguistic phylogenies: Indo-European, Austronesian, and Turkic. These large families, well represented in GeLaTo, span different regions, cultures, and histories and represent different degrees of genetic cohesiveness (Fig. 2).

### Linguistic Time Divergence Distances for Single Language Families.

**Fig. 4.**
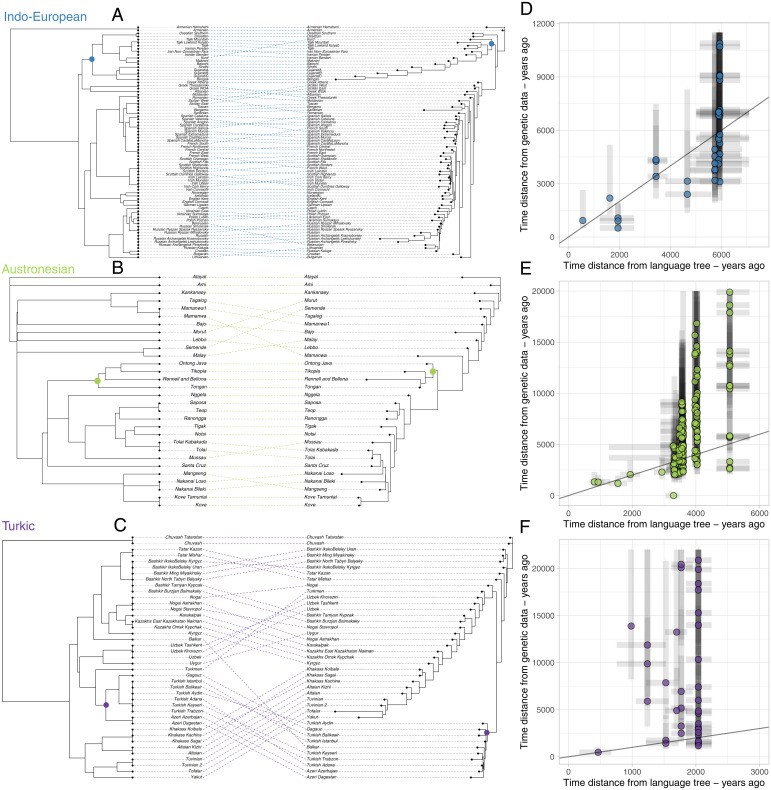
Genetic and linguistic relatedness within three language families: Indo-European, Austronesian, and Turkic. (*A*–*C*) Comparison between a linguistic tree (*Right*) generated with Bayesian models of cognate replacement (34, 48, 49) and a genetic tree generated with *F_ST_* distances (*Left*) for each language family. Each taxon corresponds to a population of the database; in some cases, the same language is spoken by different populations. In the trees, the colored dots mark clades which include the same populations in both trees: Indo-Aryan in Indo-European, Polynesian in Austronesian, and Nuclear Oghuz in Turkic. (*D*–*F*) correlation between linguistic and genetic divergence time (for the pairs for which it is possible to reconstruct effective population size). Gray bars mark 95% credible intervals from linguistic trees and 5 to 95 percentile intervals associated with the genetic divergence time reconstructions. The black line marks a 1:1 correspondence. Genetic outliers have been excluded, namely, Sardinian, Rennell and Bellona, and Mamanwa, which return very ancient divergence times (possibly due to drift and/or ancient admixture).

To zoom in on within-family comparisons, we extracted linguistic divergence times from language family trees estimated with Bayesian models of lexical replacements (*Materials and Methods*). Fig. 4 *A*–*C* provides a direct comparison of gene and language trees, in which each terminal node is a population. We estimated overall similarities in the tree structures using quartet distance between tree pairs. The highest similarity metric is found for the Indo-European trees (0.68), followed by the Austronesian (0.65) and Turkic (0.57) trees (*SI Appendix*, Fig. S23). Specific clade correspondences are found in all families, also in the Austronesian and Turkic trees, which are genetically noncohesive (Fig. 2). For example, the Polynesian branch and the Nuclear Oghuz branch are represented by genetically related groups (nodes are highlighted with a dot in Fig. 4 *B* and *C*).

We also compared linguistic and genetic divergence times for each population pair (Fig. 4 *D*–*F* and see *SI Appendix*, Fig. S24 for an alternative analysis showing mean genetic divergence times associated with each node). Vertical parallel inheritance is represented by a 1:1 correspondence (the solid diagonal line in Fig. 4 *D*–*F*). The Indo-European profile shows the highest number of pairs close to this correspondence. The overall genetic divergence timing agrees with the long-term chronology of the family supported by the linguistic phylogeny (34, 35). Old events tend to be associated with linguistic–genetic time divergence ratios close to 1 (e.g., at the root of the Indo-Aryan clade and at the split between Celtic and Romance branches; see *SI Appendix*, Fig. S24). By contrast, the Austronesian tree is characterized by an overall older divergence time for the genetic estimates, except for the Polynesian branch, where genetic and linguistic estimates are closer (11). The Turkic tree does not show relevant correspondences in the divergence times, with genetic divergence time much older than the linguistic divergence time.

## Discussion

The GeLaTo dataset allows an in-depth assessment of Darwin’s idea that the phylogenies of people and their languages coincide (2). While we caution that available genetic data are still far away from well-balanced global coverage, and that our knowledge of language relatedness remains incomplete, some suggestive patterns emerge.

At the level of individual populations, we estimate more matches (matching enclaves or aligned populations) than mismatches, but single-population mismatches are present in each continent and within each language family. Mismatches are, therefore, not exceptional events but rather regular outcomes of human history. The most common pattern among mismatches is the genetic enclave, where a population shifts to the language of a geographically neighboring but genetically distant population (Fig. 1*B* and also *Upper Left* in Fig. 2). Language shift of this kind can occur via several routes (36). For example, speakers might shift, or be forced to shift, to the language of a culturally and politically dominant population, or adopt a language which is useful for trade or cultural exchange. Sometimes, populations adopt the language of another population after genetic admixture, as in the case of Maltese. In this process, speakers from two (or more) groups in contact might bring along words, expressions, and grammatical structures from their original languages (36).

In contrast to genetic enclaves, linguistic enclaves are much rarer in our data. The only case we found is Hungarian, where speakers maintained linguistic identity despite genetic convergence with their neighbors. In this case, ancient DNA suggests a genetic replacement after the initial migration of speakers from Asia (22). While the scarcity of linguistic enclaves might be an artifact of our data or heuristics, regional studies of ancient and modern DNA suggest that it might be rare for a language to persist when its speakers assimilate genetically, or in cases of strong local genetic replacement (11, 37, 38).

At the level of language families, we find that about half of those for which we have sufficient data are genetically cohesive. Genetic cohesiveness, defined as a tendency for closer genetic distances between populations of the same language family, is in line with demographically induced spreads as proposed by the farming/language dispersal hypothesis (8, 9). However, even where phylogenies are genetically cohesive, we find that divergence times can differ (Fig. 3). In a minority of cases (Afro-Asiatic, Daghestanian, and part of Uralic), linguistic time depths tend to be deeper than genetic divergences. This temporal mismatch may result from sustained contact long after their linguistic differentiation, similar to some cases within Indo-European in Europe, a region which exhibits a substantial amount of genetic relatedness (*SI Appendix*, Fig. S2).

While deeper time reconstructions suffer from uncertainty, in particular due to our rough genetic divergence times, as explained in *Materials and Methods*, mismatches can be also be assessed at shorter time scales (Fig. 4). This reveals a variety of dynamics that can drive temporal mismatches. In some cases, genetic diversification can be a trigger for later cultural and linguistic diversification [as it has been described, for instance, in the Central Andes (39)], while in other cases, linguistic diversification can occur earlier and provide a barrier to gene flow [as suggested for Europe (7)]. Deep genetic divergence times can be the result of admixture with genetically divergent substrates. This has been suggested for populations from the early branches of the Austronesian family in near Oceania, which have been admixing with groups who migrated in the region at least 50,000 y ago and who carried a genetic ancestry sometimes referred as “Papuan” (11, 40). This early diverging genetic component in the history of the Austronesian expansion explains the very deep genetic divergences between speakers of the language family. As our method has a limited power to infer events on the deeper time scale, local comparisons of genetic and linguistic divergence should be further explored with genetic demographic simulations.

Our analyses of the GeLaTo dataset make clear that genes and languages display dynamics as diverse as the histories of the language families surveyed. Intriguingly, the family with the closest match between genes and languages is the one that has been most extensively studied and that was central in the early theorizing of gene–language correspondence: Indo-European. It remains to be seen whether this family is an outlier or reflects a more common pattern. More in-depth research on other language families is needed to move away from overrepresented regions in gene–language studies.

In conclusion, the global overview provided by GeLaTo addresses a void in anthropological studies by exploring the incongruence between genetic and linguistic diversity. Inevitably, our understanding of human history and current diversity depends on our ability to distinguish the different transmission modalities in place. A deep historical, anthropological, and linguistic contextualization of the genetic dataset is necessary in order to anchor the study of our demographic and cultural trajectories in real historical events.

## Materials and Methods

The database for this study is GeLaTo (https://gelato.clld.org/), a panel of genetic diversity with linguistic identifiers. The genetic data analyzed consists of 597,573 SNPs typed with the Human Origins Array, an SNP chip designed to minimize the effect of ascertainment bias in worldwide human diversity (41). The genetic data are collected from different publication sources (*SI Appendix*, *SI Text*). We included only populations with a minimum of five individuals for a total of 397 populations and 4,030 individuals, and we excluded sex chromosomes to not bias the analysis with the female to male ratio (*n* = 593,124 SNPs used). All the genetic populations considered are matched with a unique Glottocode identifier (42), which represents the main language spoken by the population. Linguistic relationships (i.e., language family of the spoken language per population) are based on the Glottolog classification of the world’s languages (42). Our first set of analyses consists of comparisons within and between language families. A language family is a set of languages shown to stem from a common ancestor based on the comparative method in linguistics (43). This involves showing that the languages in question have similarities that exceed chance and cannot plausibly be explained by language contact and/or universal tendencies. Most commonly, this is achieved by showing that word forms from basic vocabulary, which tend to be relatively resistant to borrowing, can be derived from a common source via regular sound correspondences.

Genetic distances are expressed as *F_ST_* distances, widely used in population genetics to quantify the genetic relatedness between populations (20). Divergence time between two populations (as generations ago) is extrapolated from *F_ST_*, being proportional to the effective population size *N_e_* with a formula equivalent to time = 2*N_e_* × linearized *F_ST_* (44). Divergence time in years is calculated with a generation time of 29 y. Dataset screening and *F_ST_* distances are calculated with PLINK (45). *N_e_* is calculated with IBDNe (46), which is based on identity by descent block coalescent. Identity by descent blocks are calculated with RefinedIBD after phasing with BEAGLE (47). We filter the results of IBDNe to keep populations with a stable size, setting a maximum threshold to exclude results biased by admixture between different ancestries, excluding populations which experienced recent expansion or collapse, and excluding variation associated with a very high CI. The variation in size is then visually screened to exclude irregular profiles. We find 164 populations as suitable to infer *N_e_*, and divergence time estimates are available only for the corresponding subset of pairwise comparisons. We consider variation in population size within the last 50 generations, due to the intrinsic limitation of the analysis methods, and assume that this would be proportional to ancestral population time in pairwise split-time reconstruction. As we do not have direct measures that go beyond this threshold, the reconstruction of ancient divergence time beyond ∼1,500 y ago is subject to further uncertainty.

Linear and smooth (generalized additive) regressions, neighbor-joining trees, quartet analysis, and data visualization are processed in R (see *SI Appendix* for further references). Analysis based on the *F_ST_* distributions is calculated with the exclusion of 18 “drifted” populations that have both continental median *F_ST_* and global median *F_ST_* above 0.1 and therefore may have experienced genetic drift due to reduced population size and/or isolation. Dataset S1 collects relevant information associated with each GeLaTo population considered, together with their genetic characterization and diversity values: the median *F_ST_* with all the other populations of the dataset, the median *F_ST_* within macro regions, the number of neighbors from a different language family within the 1,000-km radius, the lowest percentile of the *F_ST_* distribution associated with a mismatch, the closest *F_ST_* between and within language family, the associated geographic distance, and the median of the *F_ST_* between and within language family. The difference in the medians between and within language families is also annotated with relative CI. Each population is finally flagged as an enclave, a matching enclave, or as misaligned in their *F_ST_* distribution. Dataset S2 collects pairwise distance measures (i.e., genetic distance, geographic distance, divergence time). We compared *F_ST_* distances between all pairs of a given language family and between language families. Linearized *F_ST_* [equivalent to *F_ST_*/(1 − *F_ST_*)] is used for this analysis, following the IBD correlational hypothesis (27), as it retains a more linear correlation with increasing geographic distances. We focused on eight major language families that span large geographic regions and present linguistically unrelated neighbors. A geographic radius cutoff corresponding to the largest distance in kilometers between speakers of the same language family is applied, with a minimum of 500 km. For each language family, we displayed the general regression with all the population pairs.

Time depth of language family is calculated from historical linguistics sources, associated with broad indicative time ranges. A list of references considered is available in *SI Appendix*, *SI Text*. Additionally, divergence times with associated credible intervals for the root of major language families are reconstructed from generalized Bayesian dating (33).

Our second set of analyses consists of within-language family comparisons and includes linguistic distances, as linguistic divergence times extracted from summary trees generated through Bayesian analysis from previous publications (34, 48, 49).

Further information on the methods employed is available in *SI Appendix*, *SI Text*.

## Supplementary Material

Supplementary File

Supplementary File

Supplementary File

## Data Availability

Scripts to reproduce the analysis and figures in R data have been deposited in GitHub: https://github.com/gelato-org/MatchesMismatches/blob/main/AnalysisMaMi_2022_clean.R (50) and https://github.com/gelato-org/MatchesMismatches/blob/main/AnalysisMaMi_phylogenies2022.r (51). All study data are included in the article and/or supporting information. The database is available in Zenodo for direct download (52). For this work, previously published data were used from refs. 17 and 41, and the following sources (full references in the *SI Appendix*): I. Lazaridis et al., Ancient human genomes suggest three ancestral populations for present-day Europeans. *Nature.* 513, 409–413 (2014); I. Lazaridis et al., Genomic insights into the origin of farming in the ancient Near East. *Nature.* 536, 419–424 (2016); P. Qin, M. Stoneking, Denisovan ancestry in East Eurasian and Native American populations. *Mol Biol Evol.* 32, 2665–2674 (2015); P. Skoglund et al., Genetic evidence for two founding populations of the Americas. *Nature.* 525, 104–108 (2015); P. Skoglund et al., Genomic insights into the peopling of the Southwest Pacific. *Nature.* 538, 510–513 (2016); P. Skoglund et al., Reconstructing prehistoric African population structure. *Cell.* 171, 59–71 (2017). 9. F. Broushaki et al., Early Neolithic genomes from the eastern Fertile Crescent. Science (1979) 353, 499–503 (2016); C. Barbieri et al., The current genomic landscape of western South America: Andes, Amazonia and Pacific Coast. *Mol Biol Evol.* 36, 2698–2713 (2019); M. Lipson et al., Ancient West African foragers in the context of African population history. *Nature*. 577, 665–670 (2020); C. Jeong et al., The genetic history of admixture across inner Eurasia. *Nat Ecol Evol.* 3, 966–976 (2019); P. Flegontov et al., Paleo-Eskimo genetic ancestry and the peopling of Chukotka and North America. *Nature.* 570, 236–240 (2019).
