# Supplementary material for: A global analysis of matches and mismatches between human genetic and linguistic histories
Source: Proc Natl Acad Sci U S A. 2022 Nov 21;119(47):e2122084119. doi: 10.1073/pnas.2122084119 (PMC9704691; doi:10.1073/pnas.2122084119)
Supplement: Supplementary File [file pnas.2122084119.sapp.pdf]

PNAS

A global analysis of matches and mismatches between human genetic and linguistic histories

Chiara Barbieri, Damián E. Blasi, Epifanía Arango-Isaza, Alexandros G. Sotiropoulos, Harald Hammarström, Søren Wichmann, Simon J. Greenhill, Russell D. Gray, Robert Forkel, Balthasar Bickel, Kentaro K. Shimizu

corresponding author: Chiara Barbieri
Email: barbieri.chiara@gmail.com

This PDF file includes:

Supplementary text
Figures S1 to S24
Table S1
Legends for Datasets S1 to S2
SI References

Other supplementary materials for this manuscript include the following:

Datasets S1 to S2

1. Dataset description

We introduce the database GeLaTo - Genes and Languages Together, which provides the database for this study. The panel of populations analyzed is typed for the Human Origins Array (Affymetrix), a set of SNPs selected for population history studies and ascertained against the genomic diversity found in 11 individuals from different continents (1). For the analysis, only autosomal chromosomes are considered, to balance out the female/male ratio per population (593,124 SNPs used). The population samples included come from previously published genetic studies (1–13). We included only populations with a minimum of 5 individuals for a total of 397 populations and 4030 individuals with a minimum of 550,000 SNPs successfully genotyped. Missing data is ~0.1%.

All the genetic populations considered are matched with a unique Glottocode identifier (14), which corresponds to the main language spoken by the population. This information is recovered after screening the original genetic publication, and it is extrapolated either from direct sampling observation, cultural/linguistic self-identification, or geographical characterization. The proposed Glottocodes are checked by linguists and anthropologists (for a list of people who contributed expertise, see the Acknowledgments section in the main text). Populations who mainly speak a language introduced during colonial ages (widely diffused trans-national languages) are not considered for this analysis, to exclude the wave of historical language shift documented in the past ~2 centuries and keep the results conservative. Linguistic relatedness between populations corresponds to speaking languages of the same language family. Language families are therefore considered as the highest level of genealogical relatedness. Language family assignment follows Glottolog groupings. Glottolog classification is based on conservative methods that reject connections between languages not supported by strong evidence. Further historical and data-driven revisions of Glottolog, in particular on understudied regions and languages, might unveil further relatedness between languages and language families.

Our dataset contains 53 Language families and 295 languages with unique Glottocodes. Of the 53 language families, 11 are isolates, i.e. the language has no other member of its family. This is about half of the incidence of isolates in the entire Glottolog database (14), where around 40% of the language families are isolates. The reduced proportion in GeLaTo reflects the lack of genetic coverage in regions with exceptionally high cultural and linguistic diversity, such as in the Americas and Papunesia. In some instances, the same language is spoken by more than one genetic population sample. Geographic locations used through the analysis correspond to the location of the genetic populations sampled, manually curated with information from the original publications.

For this study, we use the classic Weir and Cockerham F_{ST} measurement of genetic distance between populations (15) calculated with software PLINK v. 1.9 (16) and a script available at https://github.com/epifaniarango/Fst-for-GeLaTo. Further scripts for assembling GeLaTo, with PLINK commands and data processing and screening in R (17) are available at https://github.com/gelato-org/gelato-data/blob/master/AssembleGeLaTo_2022_MaMi.R. For each population we calculate 1) the median F_{ST} between each population and any other population of the dataset, 2) the median F_{ST} between each population in their macro geographic region, and 3) the median F_{ST} between each population and their geographic neighbors in a radius of 1,000 km. Population metadata and genetic profiles are listed in Dataset S1. Pairwise comparisons between populations who live within a radius of 10 km and speak the same language have been discarded as these would produce a bias in matching patterns. These pairs of populations can be considered as duplicates, but they are in most cases genetically distinct, except in the case of the two Hungarians, two Kove and two Sulka, who share an F_{ST} = 0 with each other. As they correspond to distinct sampling studies, and they could display slightly different genetic characterizations, we do not merge the pairs into a single population.

The divergence time between two populations (as generations ago) is proportional to the F_{ST} and the effective population size (N_e) of the ancestral population with a formula equivalent to Time = $2N_e$ * Linearized F_{ST} (18). A variation of this equation implemented in (19) is also considered: the two estimates return corresponding results (Fig. S1A). For the rest of the analysis, the formula from Nei is used. Divergence time in years ago is calculated with a generation time of 29 years. To

calculate this approximate divergence time, we need an estimate of the ancestral effective population size N_e before the split. This value is calculated from the present time N_e of the two populations. Present time N_e, in turn, is often calculated as proportional to heterozygosity. Sparse SNP data, such as the one utilized in the present study, do not adequately cover the invariant sites of the genome, and therefore cannot yield an absolute heterozygosity value. To overcome this issue, here we utilize an approach based on Identity by Descent blocks, which are shared by individuals as inherited from a common ancestor. From the size and the number of Identity by Descent blocks it is possible to reconstruct the number of shared ancestors and infer variation in N_e through time: this rationale is utilized by the software IBDNe (20). Identity by Descent blocks are retrieved after phasing the data with Beagle and running refinedIBD and its associate tools for gap removals (21). IBDNe is run over the output of refinedIBD, using the blocks shared within each population. The harmonic mean of all the N_e from the last 50 generations is used to approximate N_e (this would minimize the effect of increase or decline in the last 10-20 generations). Populations with an N_e >10,000 are not considered, as such exceptionally high N_e can be resulting from population substructure. N_e values are kept only if the reconstructed variation of N_e is relatively stable in time, without a large increase or decline. Populations having very large confidence intervals associated with their N_e were then further excluded, leaving 164 populations that can be used for N_e estimations and calculations of pairwise divergence times.

To calculate the ancestral population size N_e, we average the N_e of the two populations. A second calculation is performed which uses the harmonic mean of the two N_e, which is smaller than the arithmetic mean and more affected by small values. This second formula aims at balancing the result in case one of the two populations has a very large N_e. The divergence times calculated with the two ancestral N_e reconstructions are compared in Figure S1B. Pairwise distances (genetic, geographic, and divergence times) are annotated in Dataset S2.

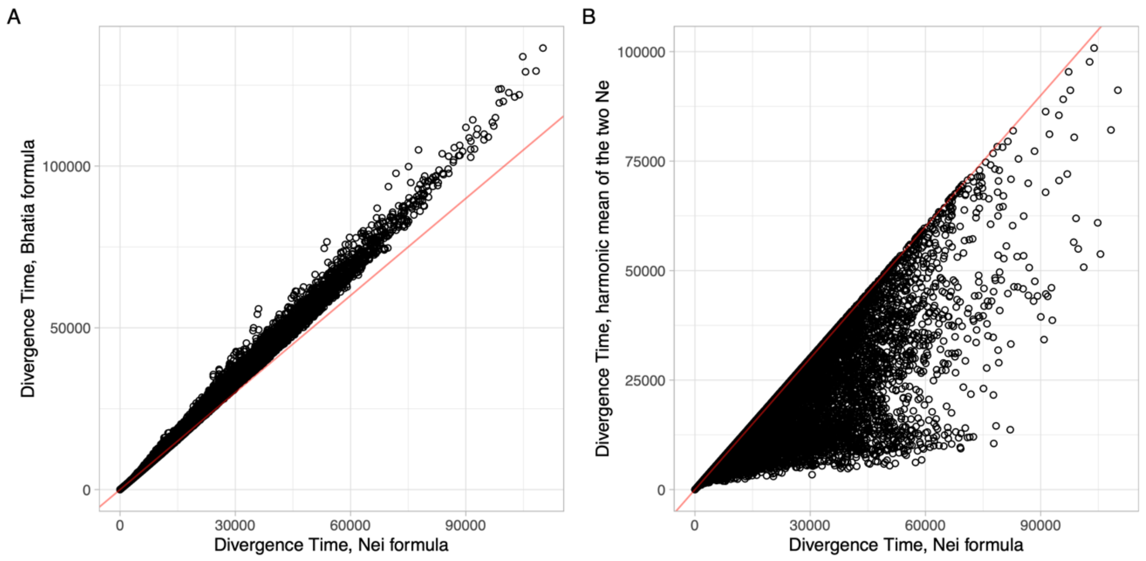

Fig. S1. Different formulas for genetic divergence time. The red line indicates a 1:1 correspondence. **A.** Comparison between two formulas considered to calculate divergence time from linearized F_{ST} and effective population size N_e, from Nei (18) and Bhatia et al. (19). **B.** comparisons with two ways of calculating the ancestral effective population size N_e between two populations: with the arithmetic mean and with the harmonic mean between the N_e of the two populations in the pair.

1a. Overview of GeLaTo dataset: coverage, language family distribution, genetic relatedness, spatial autocorrelation, time divergences

The structure of the GeLaTo database is inspected and described by looking at networks of close relatedness, geographic coverage and representativeness of different language families, incidence of spatial autocorrelation between genetic distances and reliability of time divergence estimate. The continental structure of human genetic and linguistic diversity, and the intrinsic characteristic of database coverage, can bias the identification of matches and mismatches.

The global network of genetic relatedness is displayed on a map in Figure S2. Small F_{ST} distances are weighted according to their percentile in the F_{ST} distribution. We calculate different F_{ST} density distributions for each continent, separating Africa, the Americas, Eurasia, Southeast Asia and Oceania. We consider the F_{ST} distribution within each macro continent for distances within each continent, and the F_{ST} distribution of the whole database for distances between continents, setting a series of percentile thresholds, from 0.02% to 50%, with a pace of 0.02. Each pairwise F_{ST} distance is then assigned to the corresponding percentiles in the F_{ST} distribution accordingly. Eighteen outlier populations (with average and regional F_{ST} distances above 0.1) are considered as "drifted" populations and excluded from F_{ST} distribution and percentile calculations, and subsequent analysis based on F_{ST} distribution comparisons. In Figure S2, the smallest F_{ST} distances (belonging to the lowest 10% percentile) are displayed as lines connecting the two populations involved. A dense network of close genetic relatedness is visible in Europe, reaching also North Africa and the eastern Mediterranean. Close genetic connections over long distances are found in central Asia, along Eastern Asia, between Mesoamerica and Western South America, and in sub-Saharan Africa.

To explore the power of the GeLaTo dataset in representing linguistic diversity, for each population we count the number of neighboring populations from a different language family, within a radius of 1000 km (Fig. S3A). 85% of the populations in GeLaTo do have at least one linguistically unrelated neighbor population within this radius. The median number of linguistically unrelated neighbors is five. The highest number of linguistically unrelated neighboring populations is found in the Caucasus, the Ural Mountains, Oceania, South Africa and the Mediterranean. The availability of linguistically unrelated neighbors is then explored for varying geographic radii, from 500 to 3000 km. As expected, the number of linguistically unrelated neighbors increases linearly with larger radii (Fig. S3 panel B), with more than 98% of the populations having at least one linguistically unrelated neighbor for distances > 2000 km. To represent the linguistic diversity of GeLaTo, we also count the number of different language families within a radius of 1000 km (Fig. S3 panel C).

For the analyses associated with Figure 2, we use the Isolation By Distance (IBD) model, which predicts a correlation between genetic and geographic distances (22). This model describes a gradient of genetic distances and is opposed to a scenario of strong genetic structure between populations. Attempts to estimate the strengths of IBD usually rely on the Mantel Test, which can be biased by the effects of hierarchical population structure (23). We expect our human genetic dataset to be affected by a combination of IBD and regional substructure connected by gene flow. We explore spatial autocorrelation effects with distance-based Moran Eigenvector Maps (dbMEM)(24). The RsqAdj is 0.89, suggesting that a large proportion of the genetic variation can be attributed to spatial patterns. The first four components are shown in Figure S4. The first vector highlights a strong spatial autocorrelation in South America and Asia. The second reveals a positive correlation again in the Americas, moderate in Northern Asia, and high in Europe and North Africa. The third vector of genetic and geographic correlation finds high values in Europe, North Africa and the Middle East. The fourth vector shows spatial autocorrelation in the Americas, the Middle East, Africa and Oceania. These components of geographic and genetic correlation are strong within separate continental regions and are the result of large-scale human migrations.

F_{ST} distances have been roughly converted into pairwise split divergence times, by accounting for effective population size. Excessively high population sizes can be explained by admixture and drift: populations associated with such profiles are filtered out, as well as populations that do not display a stable population size. To mitigate the effects of high population sizes in the comparisons, we also include comparisons based on the harmonic mean of the two N_e (Fig. S1B). These

divergence times do not account for migration exchanges after population split and should be taken as an indicative temporal frame for the separation between populations. Furthermore, because our method is based on IBD blocks shared from common ancestors, the ability to reconstruct population size variation becomes less reliable after 50 generations (20): this might affect the accuracy of the oldest divergence times reconstructions. The divergence time distribution for each continent is shown in Figure S5. The congruences and limitations of our divergence time reconstructions have been examined by comparing continental profiles against available knowledge on the genetic history of the continents. Eurasia divergence times are pushed as far back as 60 thousand years ago (kya), in line with the history of colonization and dispersal after the Out of Africa event (25). The split times in the Americas are below 17 kya, also in line with accredited reconstructions of the major peopling of the continent (26). The peopling of the Oceanic region started at ~5,000 years ago, but a more ancient “Papuan” ancestry admixed in current populations (27), creating larger genetic differences over the region which we see in our dataset (such as split times up to 10 kya). In Africa, our estimates are mostly below 30 kya: this date is too recent to account for the ancient structure of the continent. Genetic studies reconstructed population splits of ~100 kya between Eastern and Western Africa, and older than 200 kya for the San hunter-gatherers (Tuu and K’xa speakers) and the rest of the continent (28). The effect of migration and contact occurring over the African continent must have affected our power to reconstruct accurate deep divergence times for this continent; as stated before, deep time split events cannot be estimated reliably with the method used. With the harmonic mean formula, divergence times are noticeably more recent for Oceania, Southeast Asia and the Americas (Fig. S5B). Within these continents, Southeast Asia and the Americas display divergence times too recent to be compatible with their colonization history. The incidence of isolated populations with extremely small population sizes in these continents might be excessively magnified by the harmonic mean.

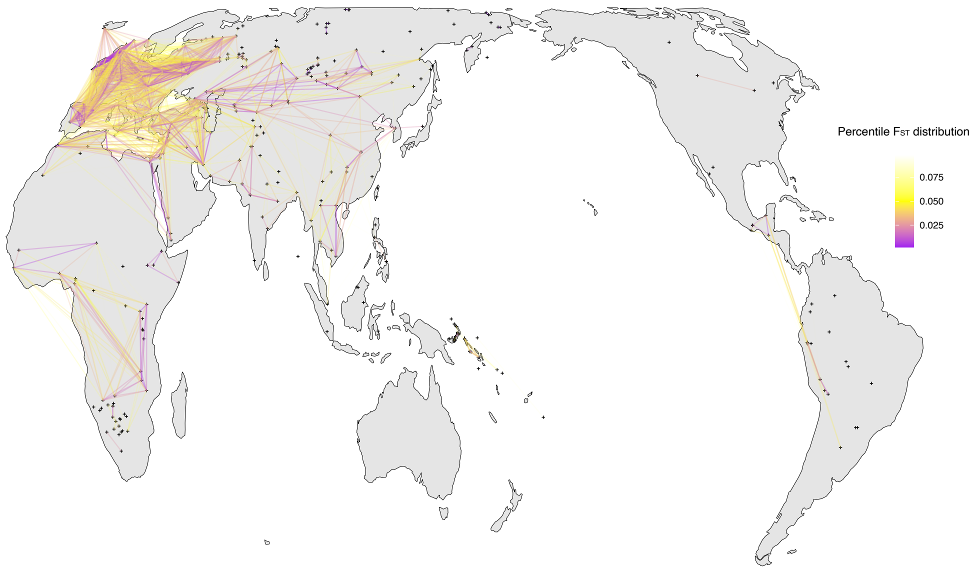

Fig. S2. Distribution of small pairwise F_{ST} distances, represented by lines connecting the two populations in the pair, and color-coded for their percentile in the global density distribution – the more saturated the color, the smaller the percentile associated with the F_{ST} distance. Only the distances below the smallest 10th percentile of the F_{ST} density distribution are shown.

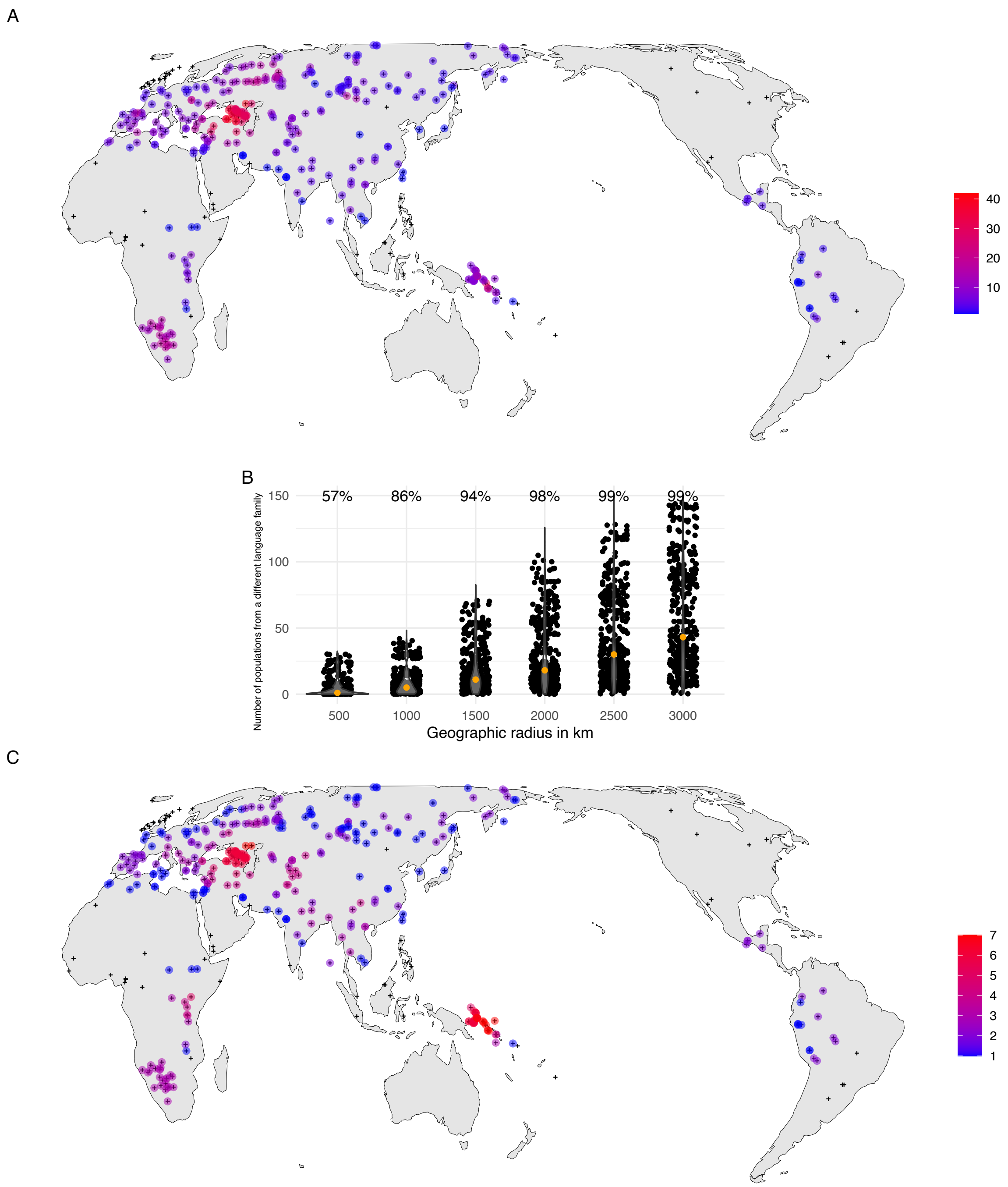

Fig. S3. Distribution of populations from a different language family within a given geographic radius. **A.** Map showing the number of populations from a different language family for each population, within a radius of 1000 km. **B.** Number of populations from a different language family using different radii, from 500 to 3000 km. The percentage of populations in the dataset which have at least one neighbor from a different language family is indicated for each radius threshold. The median is indicated by the yellow dot. **C.** Map showing the number of different language families for each population, within a radius of 1000 km.

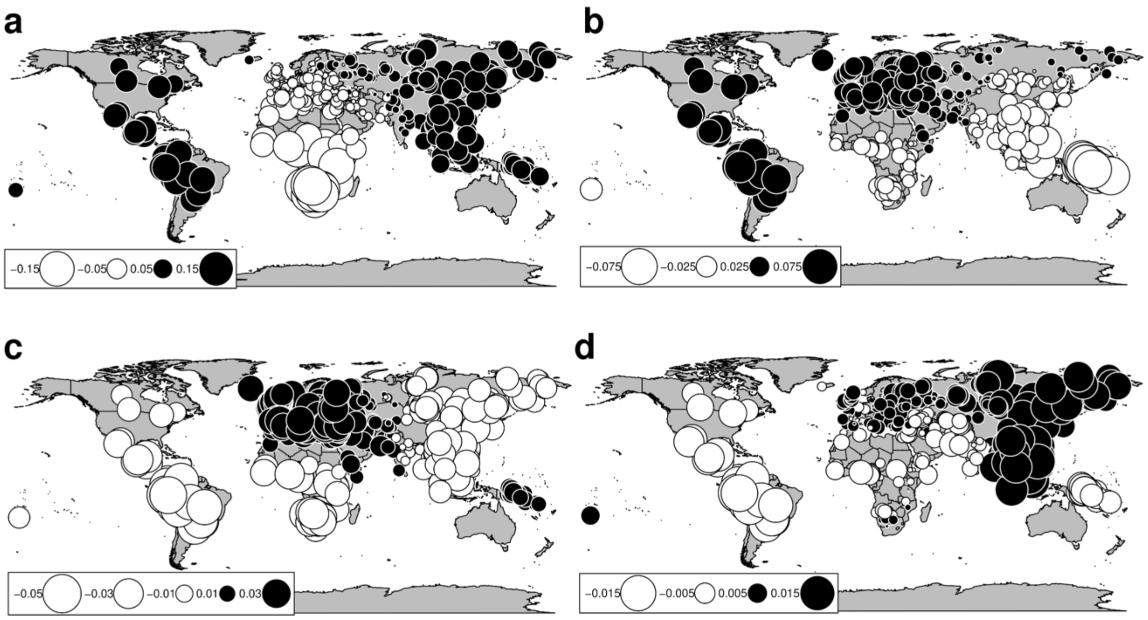

Fig. S4. Analysis of factorial dbMEM using a worldwide dataset of 395 populations associated to geographic coordinates. This method is used to illustrate spatial correlation and/or separation regarding one eigenvector (e.g., due to geographical barriers). Four levels of dbMEM eigenfunctions are shown in descending order of impact from (a) to (d). The black and white shades and their sizes indicate the difference between the different populations in relation to their position on the map. The bigger the dot the higher the positive (black) or negative (white) spatial correlation between individuals for each dbMEM eigenfunction.

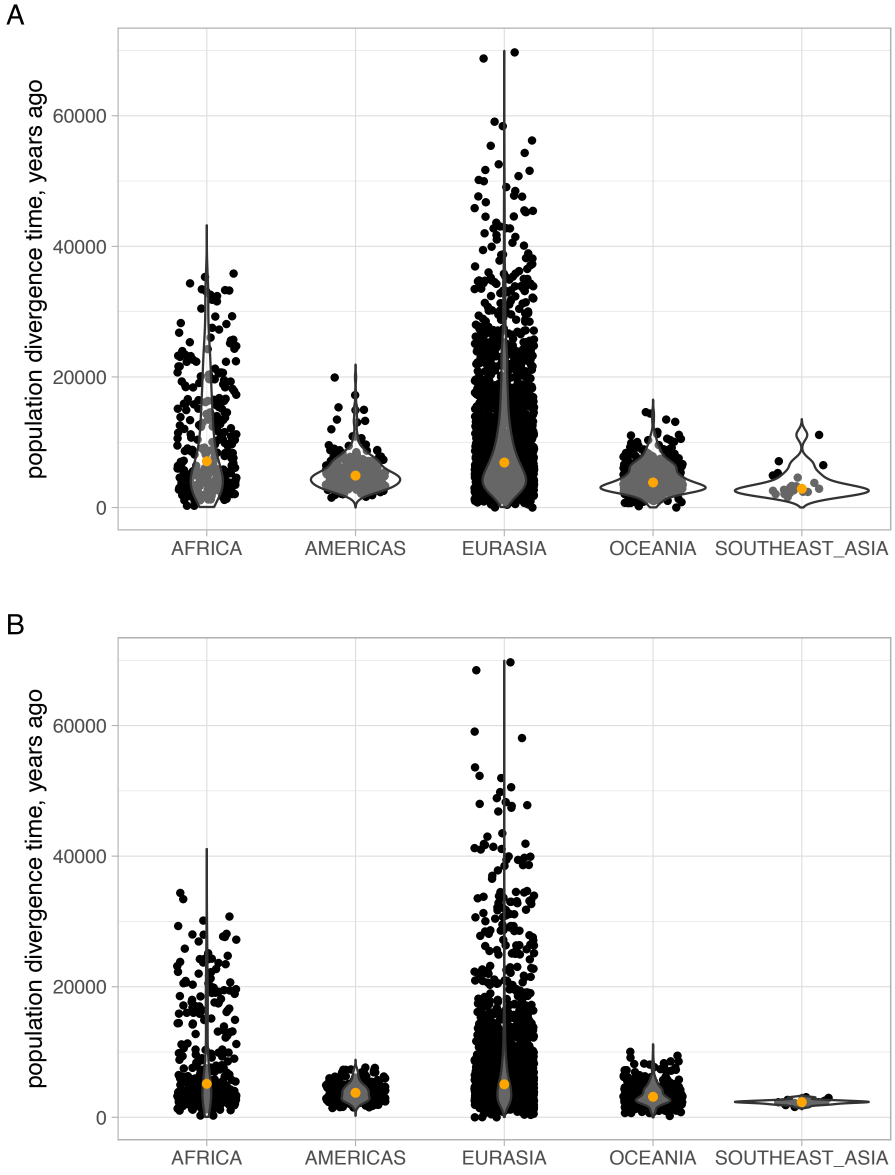

Fig. S5. Distribution of genetic pairwise population divergence time for broad geographic regions. Yellow dots indicate medians. **A.** Ancestral N_e calculated with arithmetic mean. **B.** Ancestral N_e calculated with harmonic mean.

2. Mismatches between genetic and linguistic relatedness

2a. Global overview of close genetic distances

The GeLaTo dataset is screened for pairs that are genetically close but linguistically distant, by looking at small F_{ST} distances between speakers of unrelated languages (case exemplified in Fig. S6A). Each F_{ST} distance is associated with a percentile range value in the overall continental/global F_{ST} distribution (see section above). As F_{ST} distances are differently distributed in broad macro-regions, due to different colonization processes and the Out of Africa effect, we used macro continents as distinct units of analysis for adjusting the summary statistics in global comparisons. These were shown as separate blocks of spatial autocorrelation in the main eigenvectors of the dbMEM analysis (Fig. S4). We count how many pairs have an F_{ST} below each percentile threshold. For these pairs, we count the proportion of pairs from different language families. Finally, for each population, we annotate the smallest F_{ST} percentile threshold value to a population from a different language family (Dataset S1) and for each pair, we annotate the correspondent percentile (Dataset S2).

This analysis gives a global overview of the genetic relatedness across language families (Fig. S6B). The proportion of genetically close and linguistically unrelated pairs is expected to increase with larger F_{ST} threshold percentiles. At the lowest F_{ST} threshold considered (0.02% of the distribution), 4% of the pairs speak languages from different families (Fig. S6C). This potentially corresponds to a mismatch in gene-language vertical transmission. For F_{ST} larger than the 0.14 percentile distribution, more than half of the pairs are composed of populations from different families (Fig. S6C).

Fig. S6D shows how the genetically close and linguistically unrelated pairs are distributed between 15 major language families, represented by more than 5 genetic populations. The major axes of mismatches are between language families in Eurasia: a western network includes Indo-European, Turkic, Abkhaz-Adyge and Uralic speaking populations, and a more eastern one includes Tungusic, Sino-Tibetan and Mongolic-Khitan speaking populations. These networks can be shaped by cases of population contact and episodes of shifts, which will be further discussed in the following sections.

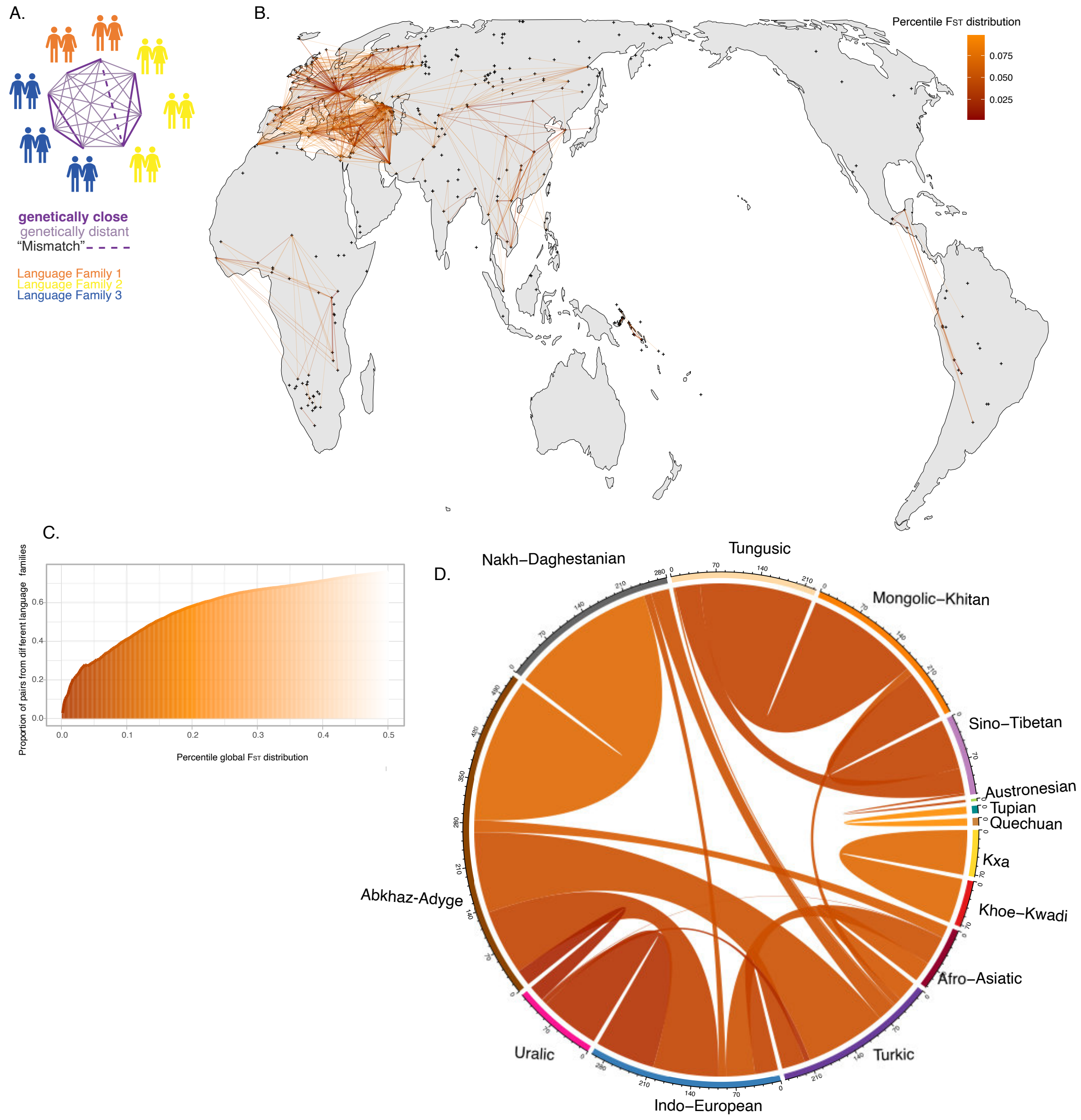

Fig. S6. Distribution of genetically close, but linguistically unrelated, pairs of populations. **A.** Schematic representation of the gene-language mismatch scenario considered. **B.** Map showing connections between pairs of populations speaking languages from different families, for different lower percentiles of the F_{ST} distribution. The darker color indicates more genetically similar populations. **C.** Histogram showing the proportion of pairs of populations that are linguistically unrelated for increasing percentiles of the F_{ST} distribution. **D.** Circle plot showing connections between genetically related pairs of populations from different language families across 15 major language families of the dataset. The total number of close genetic connections between language families (F_{ST} distances below the lowest 10% percentile) is adjusted for the number of genetic populations in each language family. The color corresponds to the median of the lowest percentile associated with the mismatches (the darker the color, the lower the median percentile of all the connections, i.e. the closer the genetic relationships).

2b. Single population mismatches: enclaves

Two heuristic descriptors are used to flag populations that show a mismatch between genetic and linguistic relatedness. The first one is a conservative analysis in which each population is tested for having a close genetic relatedness with speakers of the same language family, above geographic distance. We select only populations who belong to a language family represented in GeLaTo by more than 2 populations, and for each of them we annotate 1) the closest F_{ST} to speakers of the same language family (or same language, for the case of language isolates) and its relative geographic distance and 2) the closest F_{ST} to speakers of a different language family and its relative geographic distance. These values are reported in Dataset S1. If 2) is smaller than 1), and the population of 2) is geographically more distant than 1), the target population is flagged as presenting a "mismatch" with their linguistic and geographic neighbors and called an *enclave*. If the situation is inverted, the target population is genetically close to a geographically distant linguistic relative, and therefore is a "match" with other speakers of the same family despite the geographic distance. This is a conservative way to spot genetic migrants, who might have changed their original language to the language of their neighbors but maintained genealogical ties with the original group, which is now linguistically distant. On the other side, this test can be used to prove cases of matches that persist beyond geographic distance.

To spot the opposite case of mismatch, the linguistic migrants, we must search for populations that have very close genetic distances to their neighbors who speak an unrelated language. As seen in Figure S6, the distribution of F_{ST} varies across the continent, and it is not possible to establish a single threshold of relatedness equally meaningful for the different regions and population histories. A conservative approach is to search for populations that have an $F_{ST} = 0$ to populations from a different language family. An F_{ST} of zero would correspond to sharing the same gene pool, in complete panmixia (the variance between populations is equal to the variance between the individuals of each population). If the F_{ST} to other members of the same language family is higher, the mismatch in their linguistic affiliation is confirmed.

The 27 cases of genetic enclaves reported are listed in Table S1. Their genetically closer population from a different language family is also annotated. Only one case of linguistic mismatch/linguistic enclave can be identified: the Hungarians, here represented by two population samples, which are genetically indistinguishable from their Indo-European neighbors (29, 30). Fifty-two populations of the dataset are classified as matches above geographic distance. Twenty populations do not have a neighbor from the same language family. The population enclaves in Table S1 are ordered for geographic distance with the closest population from a different language family. The list includes the population identified as "Jew Georgian", Jewish immigrants who adopted a language from the Caucasus; Khomani, a group living in a region of South Africa where Khoe groups were dominant, but speaking a language of the Tuu family; the two populations speaking Yukagir, genetically closer to Turkic and Tungusic speakers of Siberia than to each other; Wayku, lowland Quechua speakers genetically closer to another distant Amazonian population (Cocama) than to the neighboring Andean Quechua speakers; and one Basque population from Spain, genetically closer to other Spanish speaking groups than to the neighboring Basque speaking populations of the dataset – this corresponds to a particular case applied to a linguistic isolate.

Table S1: List of enclaves.

**Population**	**Language Family**	**Geographic distance km Same Family**	**Geographic distance km Different Family**	**Different Family genetically closest population**	**Different Family genetically closest Language Family**	**Misaligned F_{ST} distribution (median F_{ST} between - F_{ST} within < 0)**	**Misaligned F_{ST} distribution (lower CI F_{ST} between - F_{ST} within < 0)**	**Misaligned F_{ST} distribution (upper CI F_{ST} between - F_{ST} within < 0)**
**Mengen**	Austronesian	121	123	Sulka Ganai	Sulka	TRUE	TRUE	FALSE
**Avar Gunibsky**	Nakh-Daghestanian	100	267	Kumyk	Turkic	FALSE	TRUE	FALSE
**Gui**	Khoe-Kwadi	187	280	Hoan	Kxa	TRUE	TRUE	FALSE
**Spanish PaisVasco**	Basque	62	341	Spanish CastillaLaMancha	Indo-European	NA	NA	NA
Hungarian1	*Uralic*	*1965*	*450*	*Czech*	*Indo-European*	*TRUE*	*TRUE*	*FALSE*
**Nama**	Khoe-Kwadi	549	589	Khomani	Tuu	**FALSE**	**FALSE**	**FALSE**
**Khomani**	Tuu	521	589	Nama	Khoe-Kwadi	FALSE	TRUE	FALSE
**Zapotec**	Otomanguean	149	632	Kaqchikel	Mayan	NA	NA	NA
Hungarian2	*Uralic*	*1290*	*646*	*German Lipsian*	*Indo-European*	*FALSE*	*TRUE*	*FALSE*
**Khwe**	Khoe-Kwadi	435	729	Tswana	Atlantic-Congo	FALSE	TRUE	FALSE
**Wayku**	Quechuan	162	768	Cocama	Tupian	TRUE	TRUE	FALSE
**Jewish Georgian**	Kartvelian	341	857	Turkish Kayseri	Turkic	**FALSE**	**FALSE**	**FALSE**
**Azeri Azerbaijan**	Turkic	1088	1260	Iran_Non-Zoroastrian_Fars	Indo-European	TRUE	TRUE	TRUE
**Bengali**	Indo-European	1208	1324	Vishwabrahmin	Dravidian	**FALSE**	**FALSE**	**FALSE**
**Han-NChina**	Sino-Tibetan	601	1325	Korean	Koreanic	**FALSE**	**FALSE**	**FALSE**
**Dai**	Tai-Kadai	803	1337	Vietnamese South	Austroasiatic	NA	NA	NA
**Hazara**	Indo-European	490	1507	Uygur	Turkic	TRUE	TRUE	TRUE
**Yukagir Tundra**	Yukaghir	584	1875	Evenk FarEast	Tungusic	NA	NA	NA
**Yoruba**	Atlantic-Congo	695	1889	Mende	Mande	**FALSE**	**FALSE**	**FALSE**
**Yaquis**	Uto-Aztecan	249	2157	Maya	Mayan	NA	NA	NA
**Mongola**	Mongolic-Khitan	1195	2345	Xibo	Tungusic	**FALSE**	**FALSE**	**FALSE**
**Evenk FarEast**	Tungusic	317	3050	Mongol Uuld	Mongolic-Khitan	**FALSE**	**FALSE**	**FALSE**
**Cocama**	Tupian	2851	3455	Maya	Mayan	TRUE	TRUE	FALSE
**Karitiana**	Tupian	1993	4457	Maya	Mayan	NA	NA	NA
**Surui**	Tupian	1863	4613	Maya	Mayan	NA	NA	NA
**Yukagir Forest**	Yukaghir	584	5835	Karakalpak	Turkic	NA	NA	NA
**GuaraniGN**	Tupian	648	6159	Maya	Mayan	**FALSE**	**FALSE**	**FALSE**
**Guarani**	Tupian	2851	6221	Maya	Mayan	FALSE	TRUE	FALSE
**Aleut**	Eskimo-Aleut	1103	6226	Bashkir North Tabyn Balysky	Turkic	NA	NA	NA

Table S1. List of populations flagged as genetic enclaves and linguistic enclaves (the latter corresponding to two Hungarian genetic populations, in italic). The last three columns show features associated with the second heuristic criteria for mismatch, the misalignment of F_{ST} distributions. NA indicates that the number of available comparisons is too small to calculate a distribution of F_{ST} within language family and between language families. If both the median and the lowest CI of the difference in between-within family comparisons are positive (highlighted in boldface), the population is in alignment with their linguistic relatives, in opposition to the status of mismatch from the genetic enclave assignation.

However, many of these single mismatch cases are non-informative. In the Americas, some populations from the Tupian or Uto-Aztecan families are genetically close to Maya, but the Maya group shares genetic similarities all over the continent, probably due to their less drifted genetic profile and/or to a proposed gene flow from Mesoamerica to the south (31). Nama speakers are also on the list, because of their genetic proximity with Khomani, but the latter is instead the one historically matching the scenario of a language shift. Nama speakers have been described as genetically similar to southern Tuu speakers, living in regions from where the Nama originally came from (32, 33). Han speakers are genetically similar to Koreans, but is rather the latter who is driving this connection because of their genetic similarity to continental Asia; furthermore, the Koreanic language family, which is a very small family represented by two languages, is not represented by any other genetic population and cannot be flagged as mismatching by this method. Finally, Bengali are genetically close to a Dravidian group but are geographically isolated, and their closest neighbor is at more than 700 km of distance, thus making the comparison less informative. These examples show the potentials and limitations of this strict search for mismatching populations, which is heavily influenced by the structure of the dataset. First, the range of geographic neighbors: if the closest geographic neighbors are too distant, the matching is not informative (cases in the bottom rows of Table S1). Second, the population from a different language family which is genetically close but geographically distant can be the “exceptional” one driving this mismatch: either because it has very close F_{ST} with many populations, even at large geographic distances (this is the case of Maya, driving most of the possible mismatches found in South America), or because is the one possibly having experienced the language shift.

2c. Single population mismatches: misaligned F_{ST} distributions

For our second screening of population matches and mismatches, we compare F_{ST} distributions within and between language families. This overview would account for a degree of overlap in the two distributions and accept a more flexible and realistic scenario. Each target population was tested for their distribution of F_{ST} distances with a) speakers of the same target language family and b) speakers of different language families. In principle, most of the populations of the dataset are expected to show a smaller F_{ST} with the speakers of their language family in comparison to the F_{ST} of all the linguistically unrelated populations all over the continents. To circumscribe the test to a similar baseline of potential genetic relatedness, a geographic maximum radius was applied to this comparison. This threshold corresponds to the maximum geographic distance between the target population and the other populations of the same language family. A minimum radius of 500 km is applied for language families with a small geographic extension. 64 populations have negative values of median F_{ST} between-median F_{ST} within, over 316 populations for which the two medians can be calculated (20%).

The highest values of alignment (higher between-language family F_{ST}) are found in Africa for the Atlantic-Congo speakers, which have high F_{ST} distance with most hunter-gatherer groups in southern Africa speaking languages from the Tuu and Kx’a families (Fig. S7-8). A high number of misaligned populations is present in the Caucasus, and a smaller number is found in Europe (Fig. S7C and D). Through Island Southeast Asia and the Pacific there is a longitudinal gradient with higher F_{ST} within the Austronesian language family in the west and smaller values in the east (Fig. S7E).

After this screening, some of the genetic enclaves discussed above are not immediately confirmed as mismatches, as they have median F_{ST} distances within language families smaller than those

between language families. In addition, 13 populations also have a positive lower Confidence Interval associated with the difference between and within language family F_{ST} (the last columns in Dataset S1). These 13 populations were previously flagged as genetic enclaves but, when placed in context, appear overall genetically close to their linguistically related populations. These 13 populations are Jewish from Georgia, Han, Yoruba, Mongola, Bengali, Nama, Guarani, Bengali, Khomani, Avar Gunibsky, Evenk Far East and one of the two Hungarian populations. Four populations previously classified as Matches qualify as misaligned under the F_{ST} distribution criteria: these are Madak, Santa Cruz, Atayal and Karachai. In contrast, 65 cases of mismatches with misaligned F_{ST} distributions are found, of which six were previously flagged as enclaves (Hazara, Cocama, G|ui, Azeri Azerbaijan, Mengen and one of the two Hungarian populations). Relevant cases of misaligned populations are those also associated with a small confidence interval. Populations with a negative difference in median F_{ST} and an associated CI < 0.01 are Maltese, Chechen, Ingushian, Tatar_Mishar, Hazara and Cochin Jews. In Africa, Naro and ǂHoan are also relevant outliers as the only population with a negative difference of medians for the Khoe and Kx'a language families, respectively (Fig. S7B, S8). These two populations have previously been described as undergoing language shifts with evidence of substantial linguistic contact and sociocultural power imbalance (2, 34).

To address the over representation of some language families, we perform a downsampling sensitivity test. We randomly selected a maximum of 8 populations per language family, and then calculated the proportion of populations:

1) close to a linguistically unrelated population,

2) matching enclaves,

3) genetic/linguistic enclaves, and

4) aligned/misaligned populations.

We repeated this procedure 100 times. The subsampled datasets of 185 populations return a proportion of matches and misaligned populations compatible with that found in the whole dataset. However, the populations close to linguistically unrelated populations and the mismatches become more numerous with the random downsampling iterations, possibly more affected by less dense population coverage (Fig. S11).

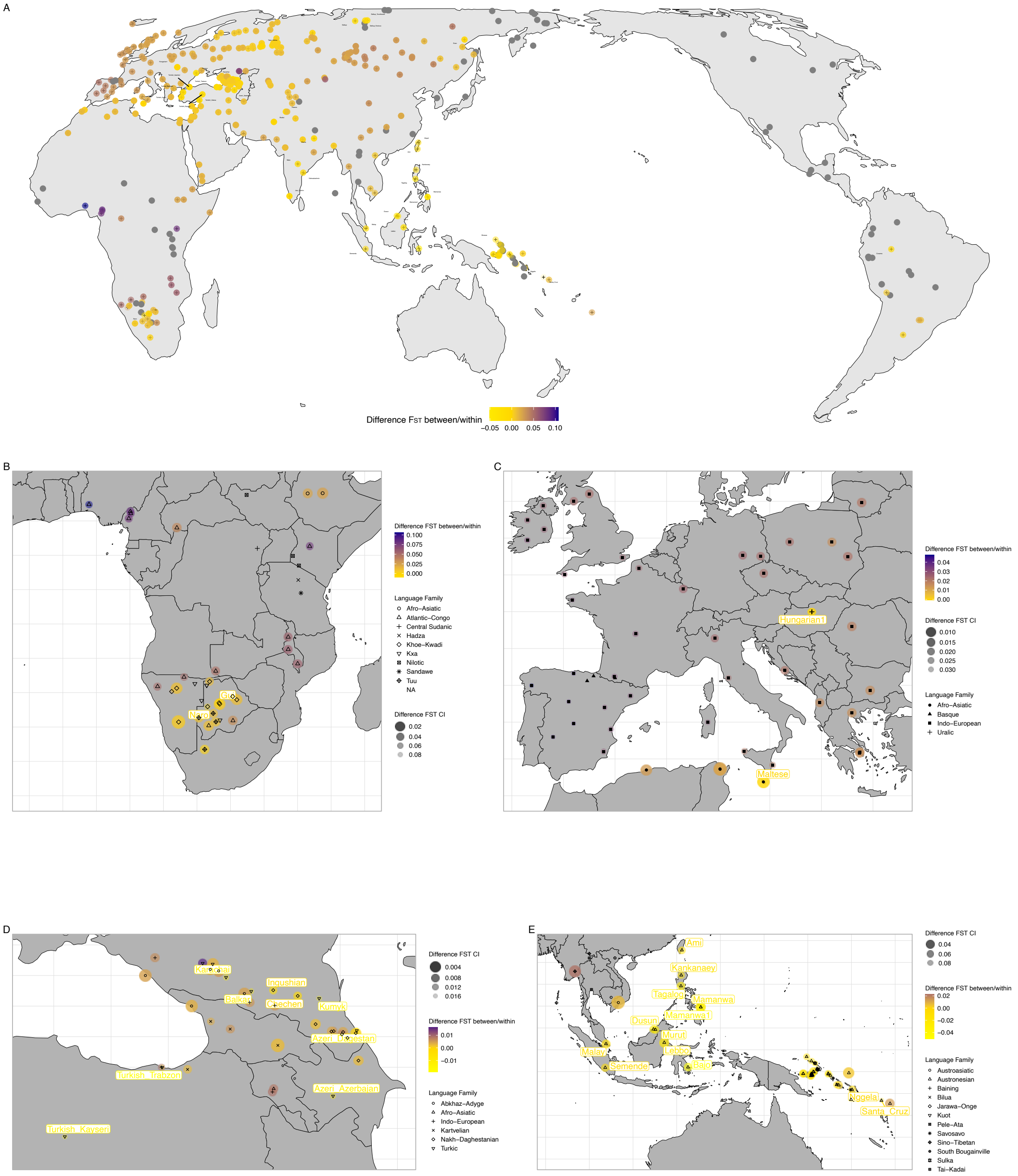

S7. Maps showing the difference between median F_{ST} values between and within language families. Yellow color marks populations in misalignment. **A.** Global overview. Population names mark populations in misalignment, for which the between language family median F_{ST} is smaller than the within language family median F_{ST}, and the CI associated with this difference is below 0.01. Transparent points have larger confidence intervals associated with the median difference. **B-E.** Regional cases discussed in the text: Africa, West Eurasia, Caucasus, Southeast Asia and Pacific.

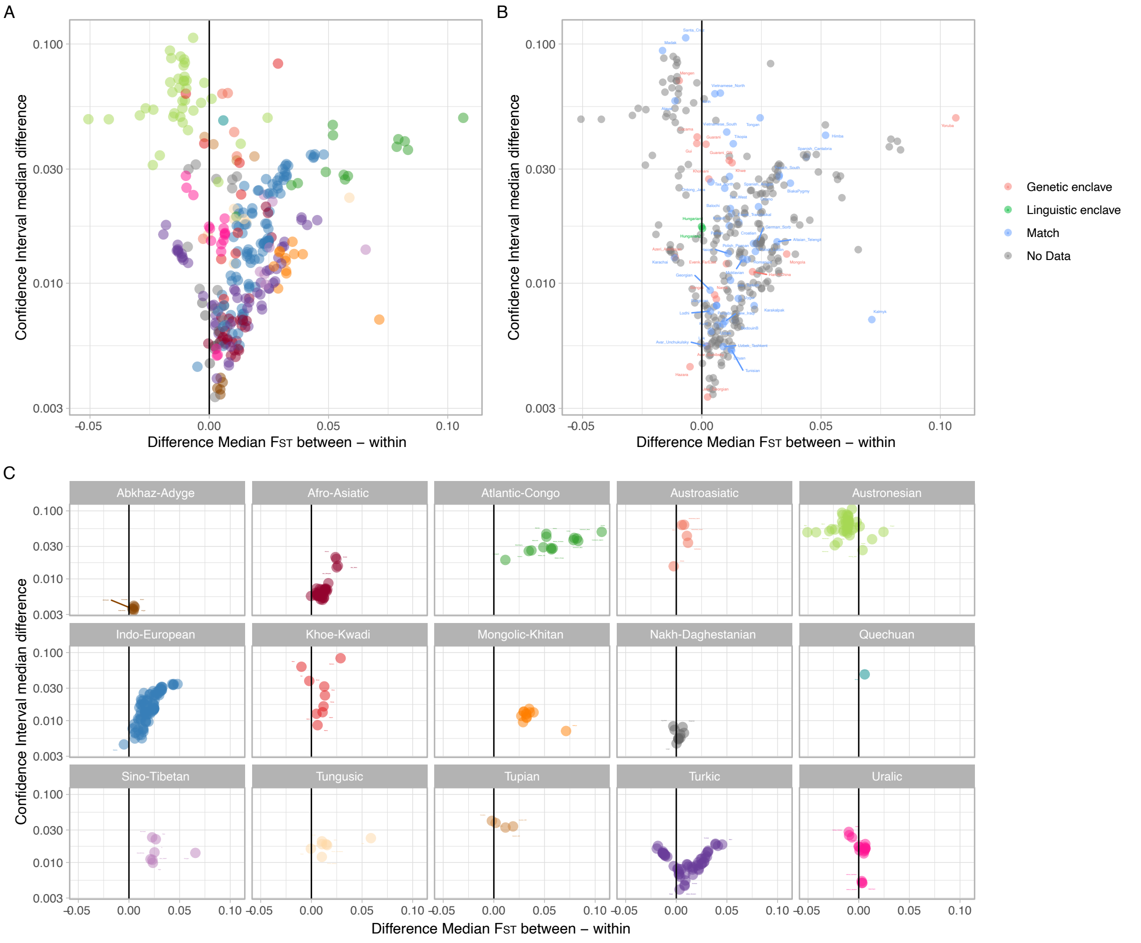

Fig. S8. Difference between median F_{ST} values between and within language family, and the associated Confidence Interval for each population. **A.** Whole dataset, with points colored by major language family affiliation. **B.** Whole dataset, highlighting populations previously flagged as genetic enclaves (red), linguistic enclaves (green), or matching enclaves (blue). **C.** Subsets of the global distribution for 15 major language families.

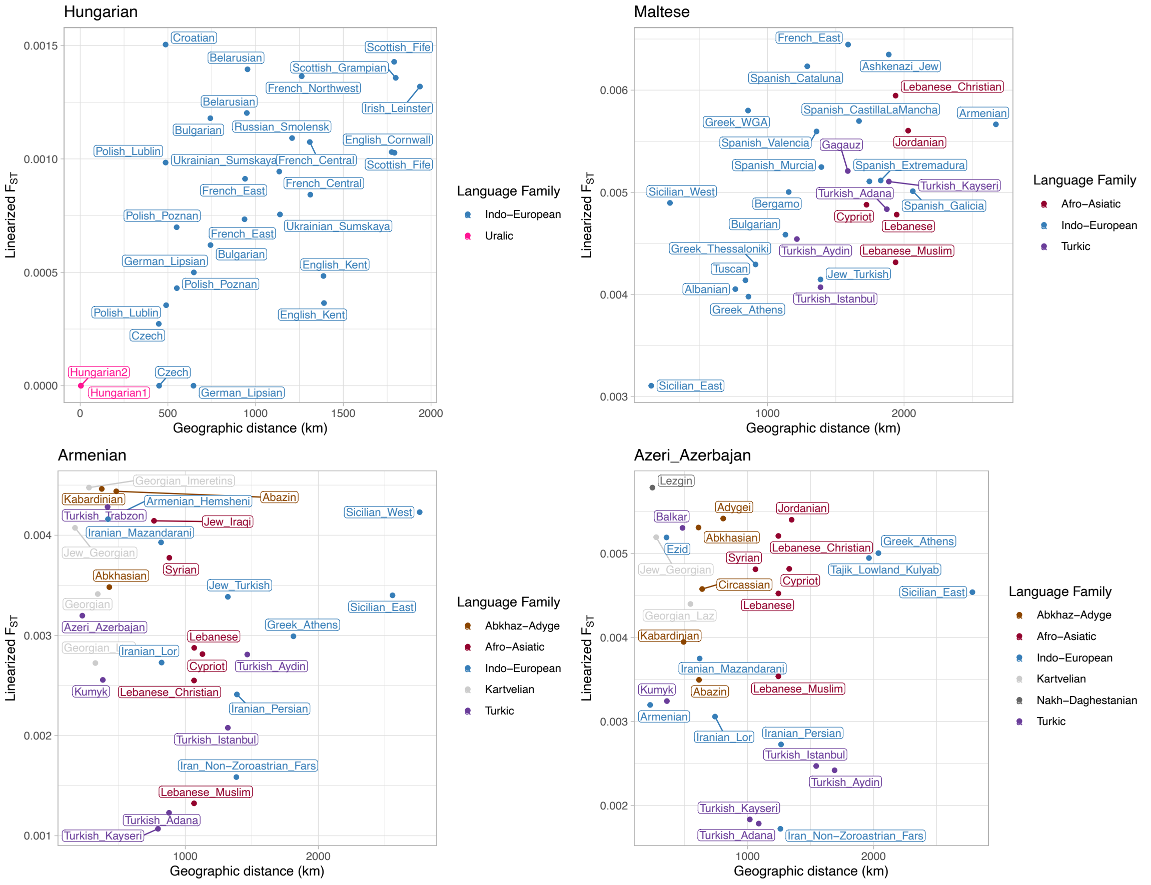

Fig. S9. The relationship between linearized F_{ST} and geographic distances for the four case study populations discussed in the main text (Hungarian, Maltese, Armenian and Azeri Azerbaijan). For each case study population, we label the 30 genetically closest populations, color coded for their language family. On the x axis, the geographic distance to the target population, in km.

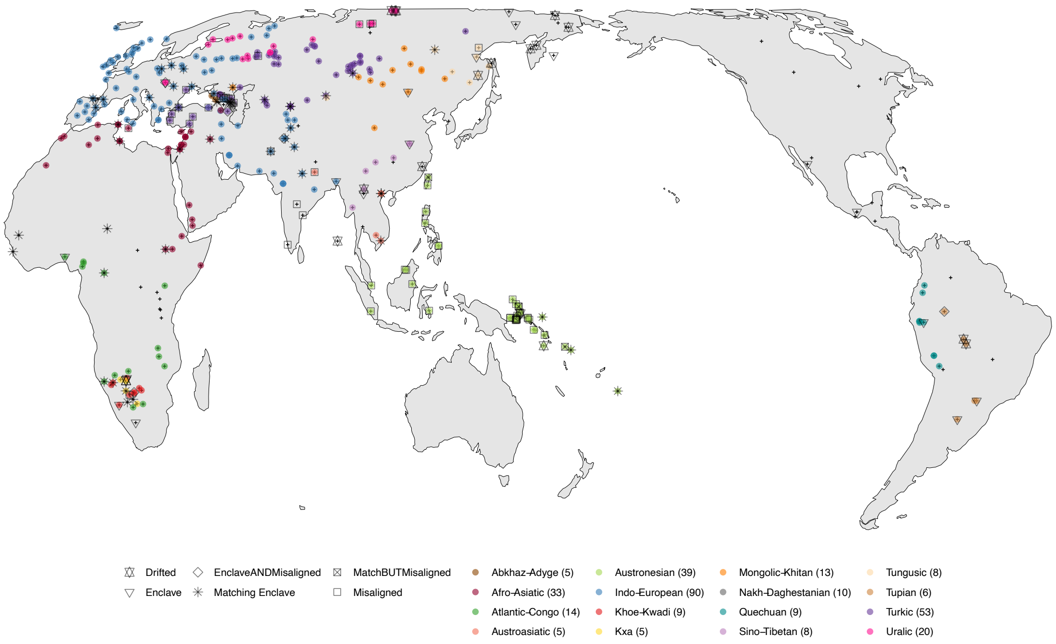

Fig. S10. Characterization of populations included in GeLaTo. The map shows the approximate location of each population, represented by black crosses. Major language families (with more than 5 populations) are color-coded with a solid circle. Larger symbols differentiate single population cases as matches or mismatches according to the different heuristic criteria employed, or as drifted (and hence excluded from F_{ST} distribution comparisons as their median F_{ST} is exceptionally high > 0.1). In the color legend, the numbers in parenthesis correspond to the number of population samples for each language family.

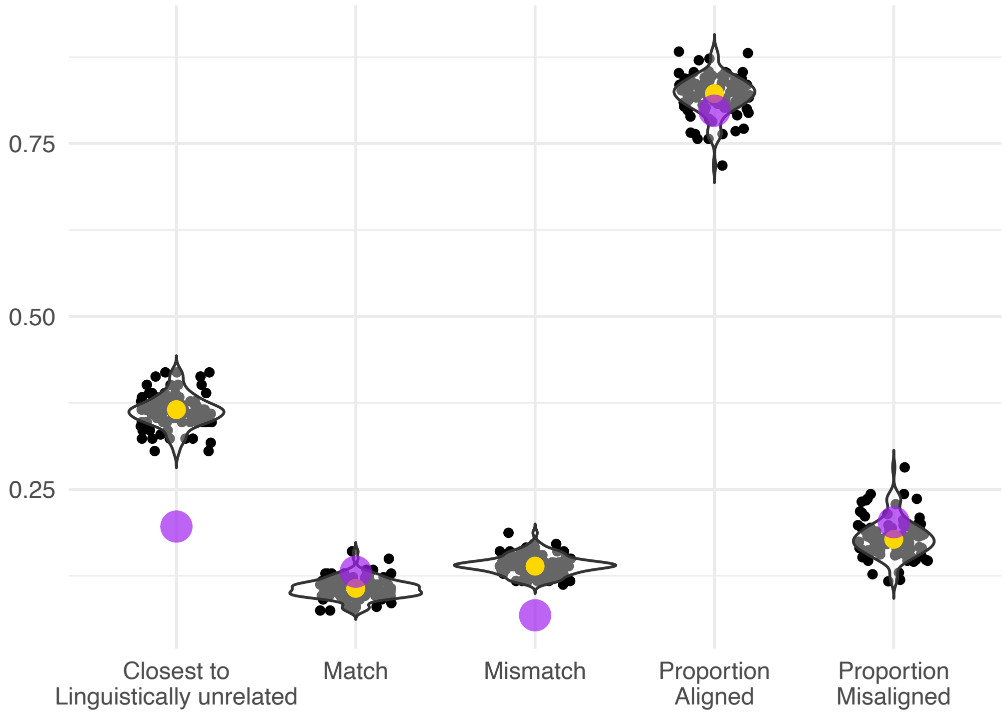

Fig. S11. Downsampling sensitivity test. The large purple points correspond to the proportion of populations from the full GeLaTo dataset. The black points correspond to the proportion of populations flagged as (1) closer to linguistically unrelated populations, (2) match, (3) mismatch, (4) aligned, and (5) misaligned, over 100 iterations of random subsamples. The yellow points correspond to the median of these 100 iterations.

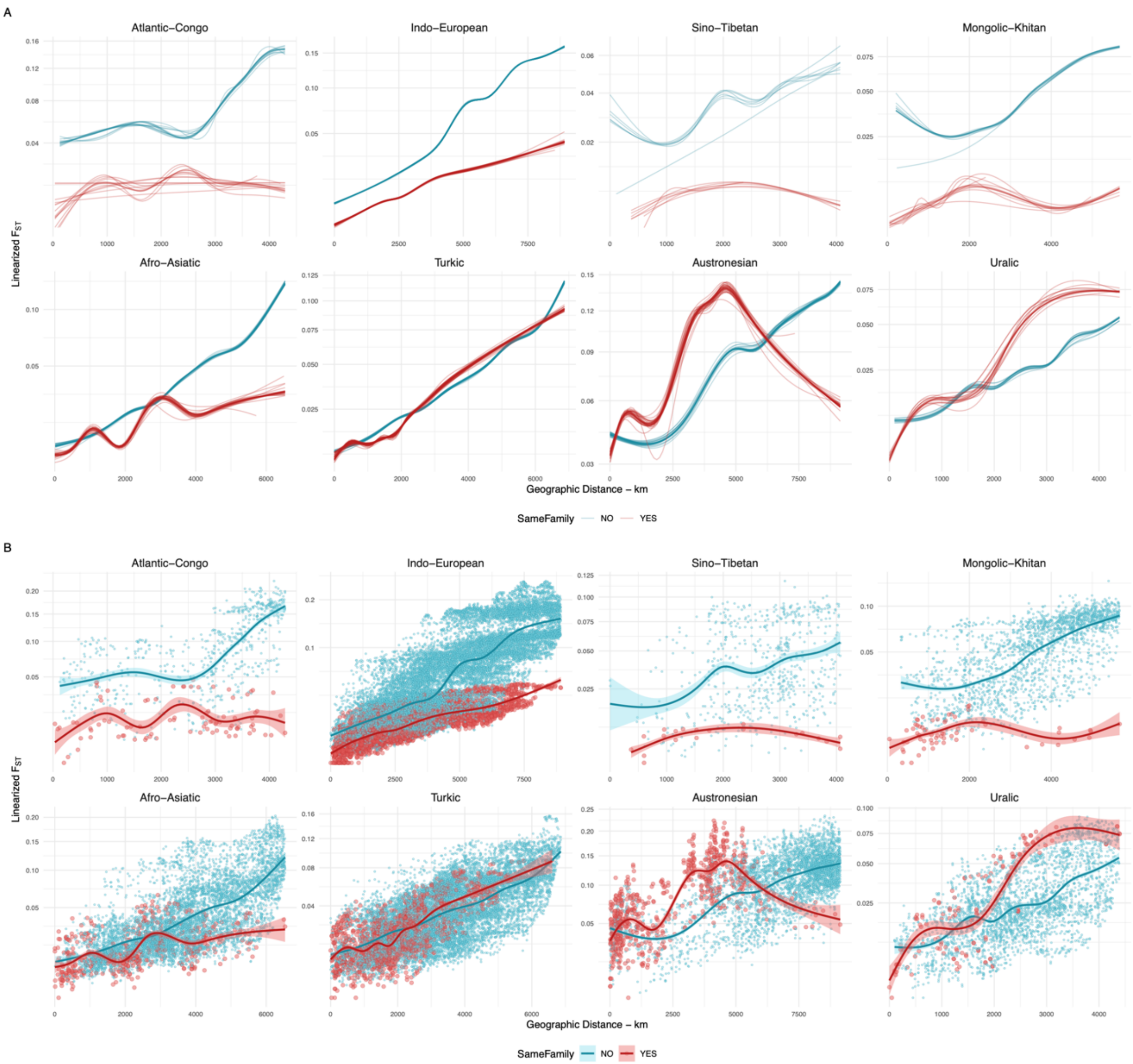

Fig. S12. Sensitivity analyses of the genetic cohesiveness of language families. **A.** Jackknife analysis of the language family comparisons at the population level. The plots show the correspondence between genetic distances and geographic distances for 8 major language families (see Figure 2). In the top row panels, language families are mostly genetically cohesive; in the bottom row panels, language families show an ambiguous profile. In each round of jackknife, we remove one population from the language family together with all pairs that included the population. Smooth (i.e., generalized additive) regressions summarize the between and within family trends for each round of jackknife. Although removing specific populations changes the regressions (see Figures S13-20 for details), none alters the overall distinctions or patterns. **B.** Smooth (generalized additive) regressions after removing the distances of those pairs of populations that have a higher influence (Cook's Distance) on the model than a 4/N threshold. Like in the population-level analysis, the overall patterns and distinctions are robust against removing these datapoints.

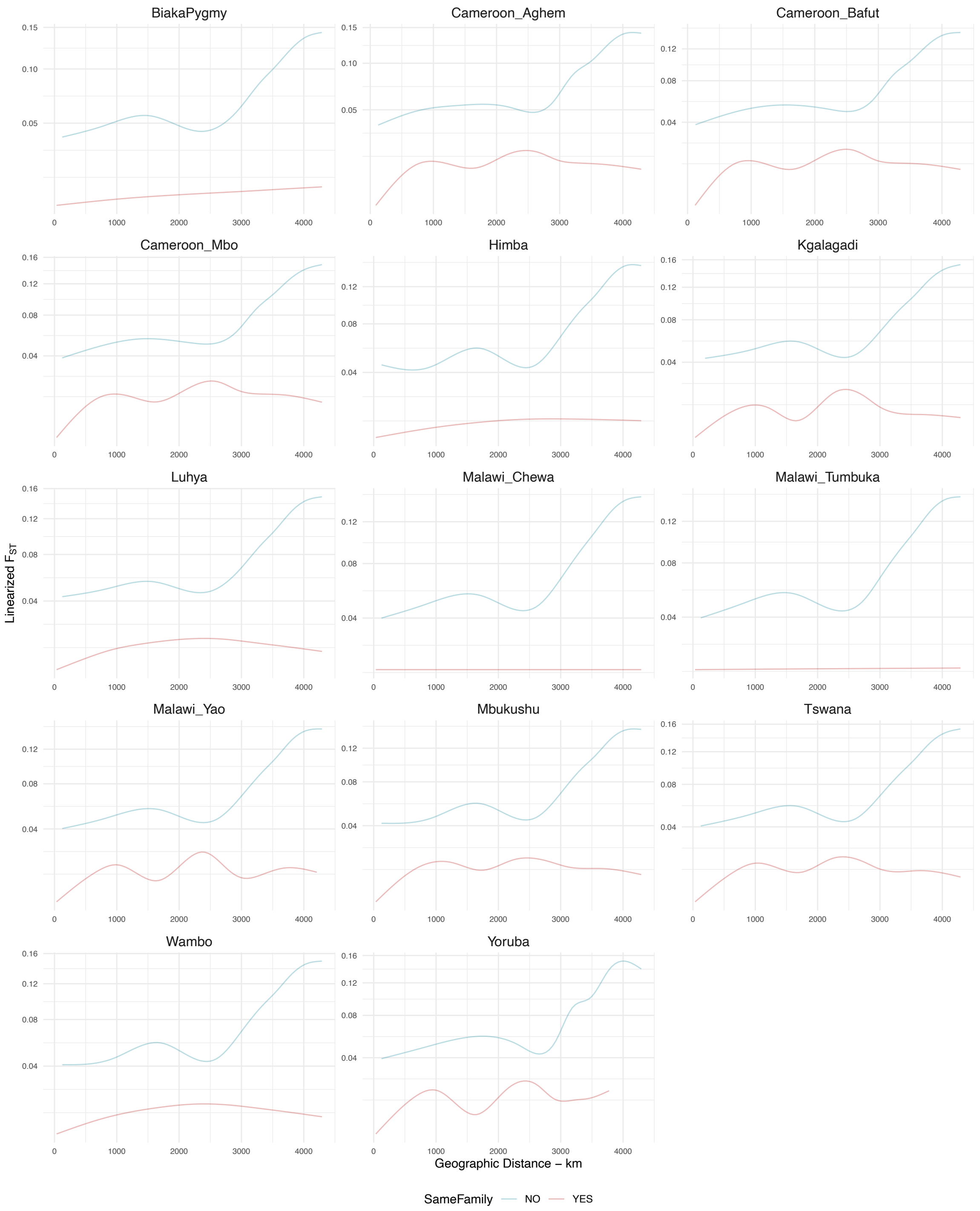

Fig. S13. Correspondence between genetic distances and geographic distances for the Atlantic-Congo language family. Smooth (i.e., generalized additive) regressions summarize the between and within family trends. In each panel, the population indicated in the title is removed from the comparisons, to show how each population from the family influences the overall pattern in Figure S12A.

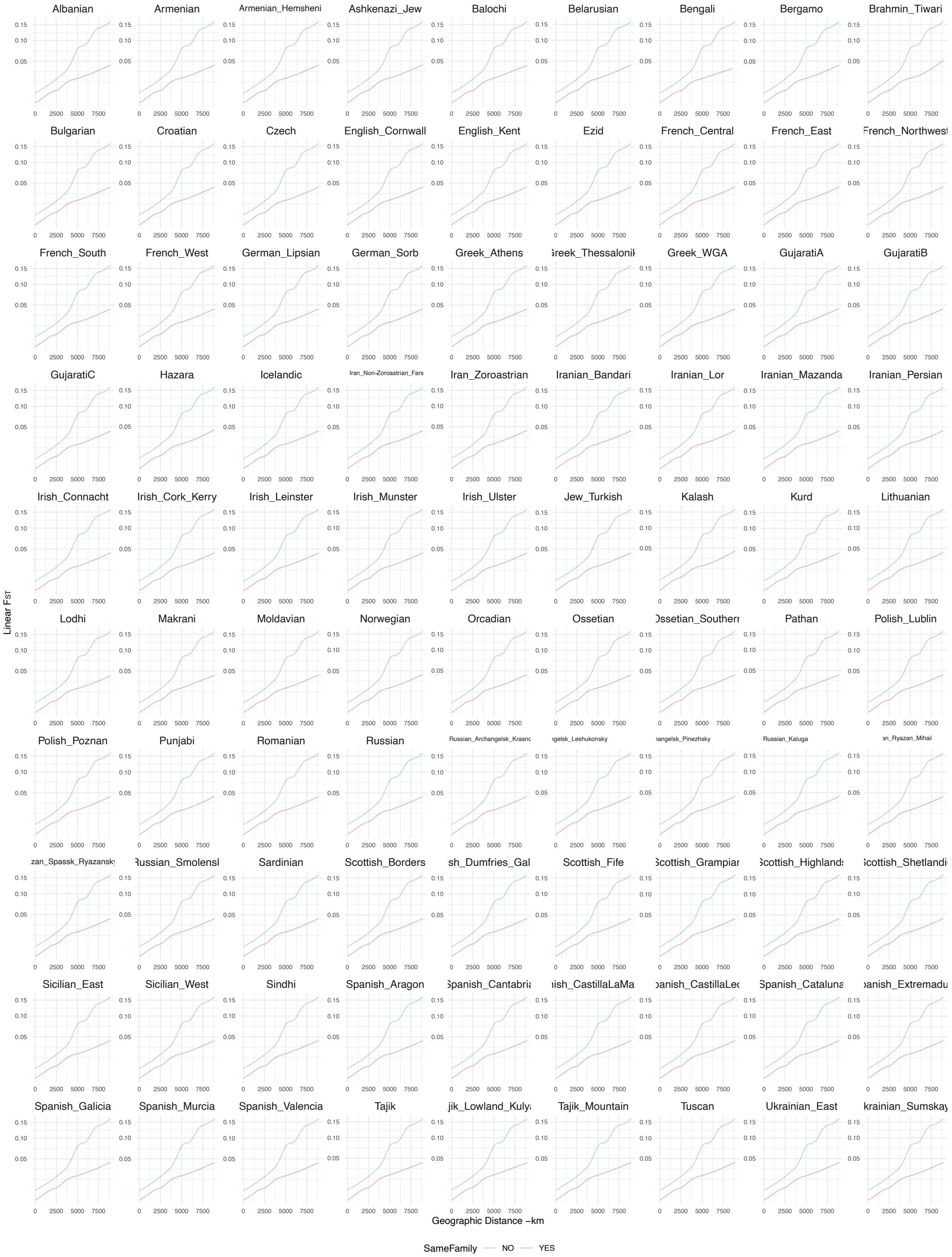

Fig. S14. Correspondence between genetic distances and geographic distances for the Indo-European language family. Smooth (i.e., generalized additive) regressions summarize the between and within family trends. In each panel, the population indicated in the title is removed from the comparisons, to show how each population from the family influences the overall pattern in Figure S12A.

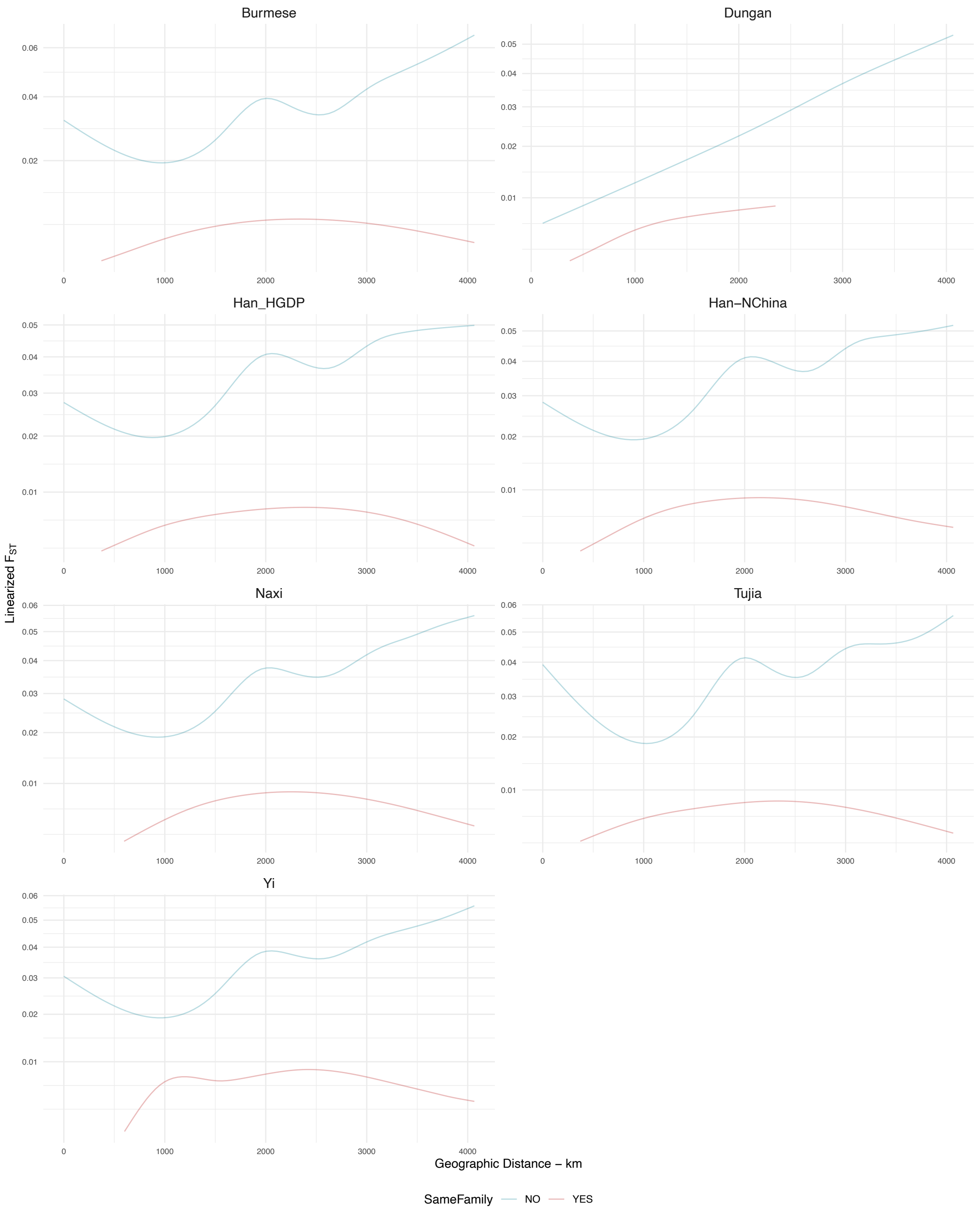

Fig. S15. Correspondence between genetic distances and geographic distances for the Sino-Tibetan language family. Smooth (i.e., generalized additive) regressions summarize the between and within family trends. In each panel, the population indicated in the title is removed from the comparisons, to show how each population from the family influences the overall pattern in Figure S12A.

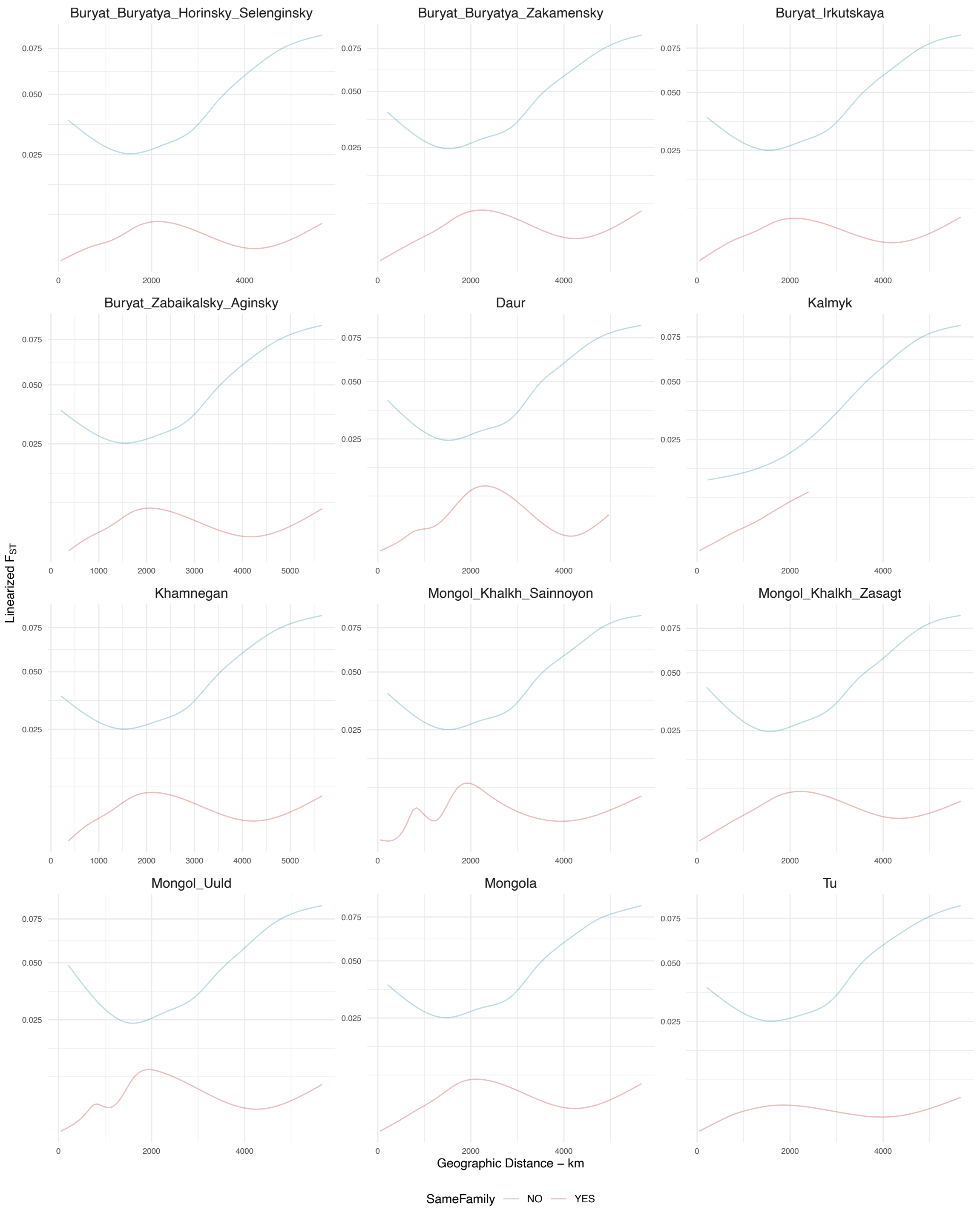

Fig. S16. Correspondence between genetic distances and geographic distances for the Mongolic-Khitan language family. Smooth (i.e., generalized additive) regressions summarize the between and within family trends. In each panel, the population indicated in the title is removed from the comparisons, to show how each population from the family influences the overall pattern in Figure S12A.

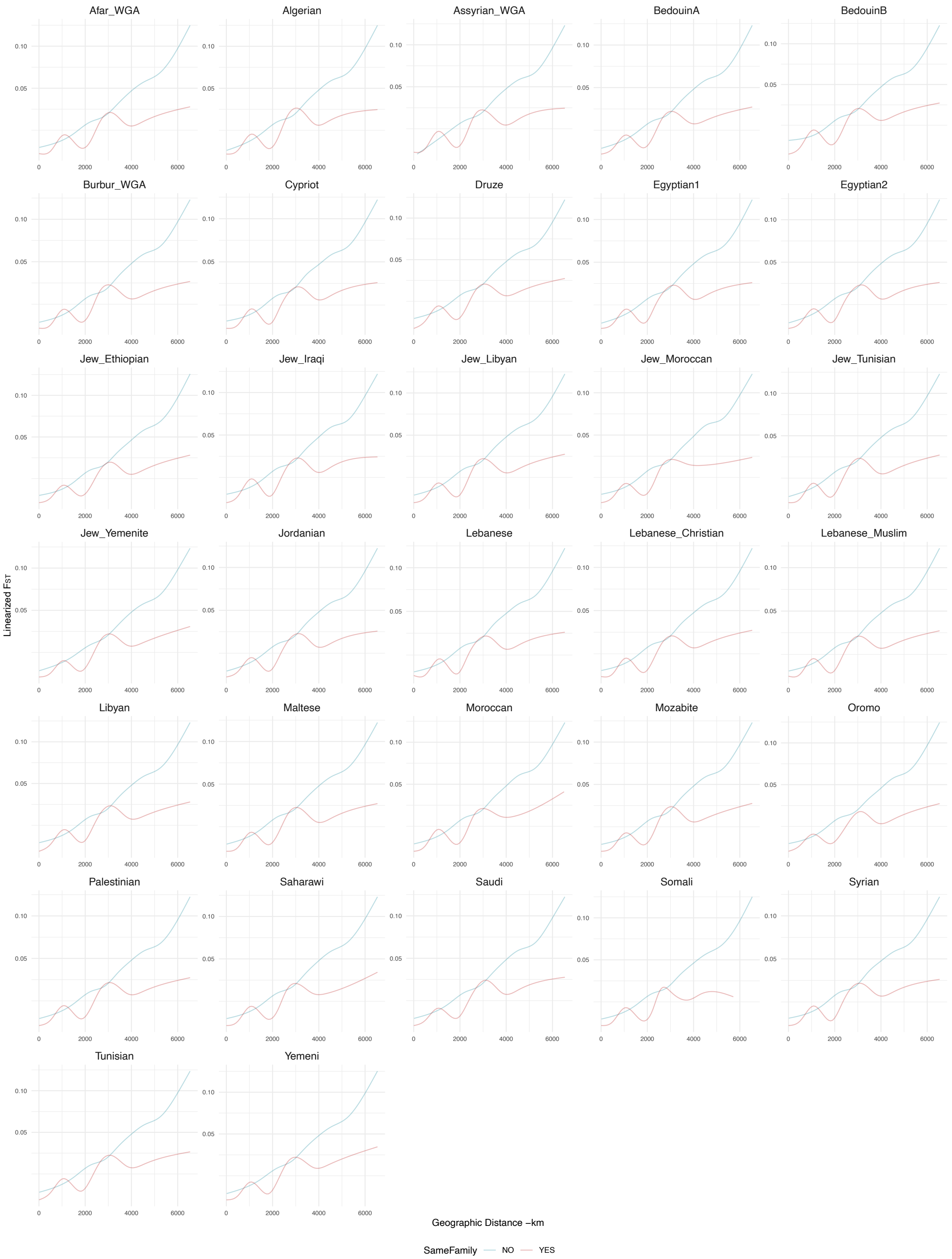

Fig. S17. Correspondence between genetic distances and geographic distances for the Afro-Asiatic language family. Smooth (i.e., generalized additive) regressions summarize the between and within family trends. In each panel, the population indicated in the title is removed from the comparisons, to show how each population from the family influences the overall pattern in Figure S12A.

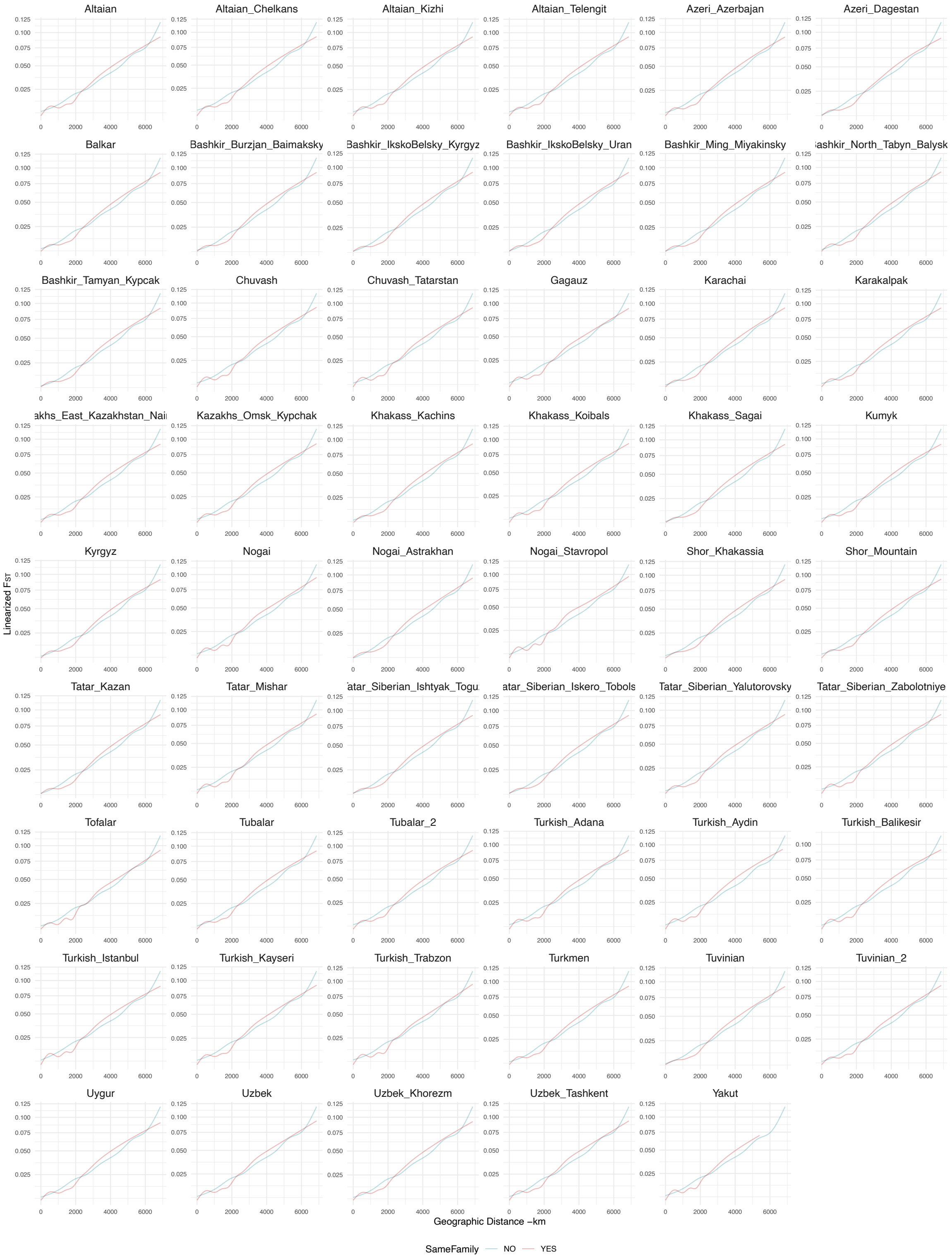

Fig. S18. Correspondence between genetic distances and geographic distances for the Turkic language family. Smooth (i.e., generalized additive) regressions summarize the between and within family trends. In each panel, the population indicated in the title is removed from the comparisons, to show how each population from the family influences the overall pattern in Figure S12A.

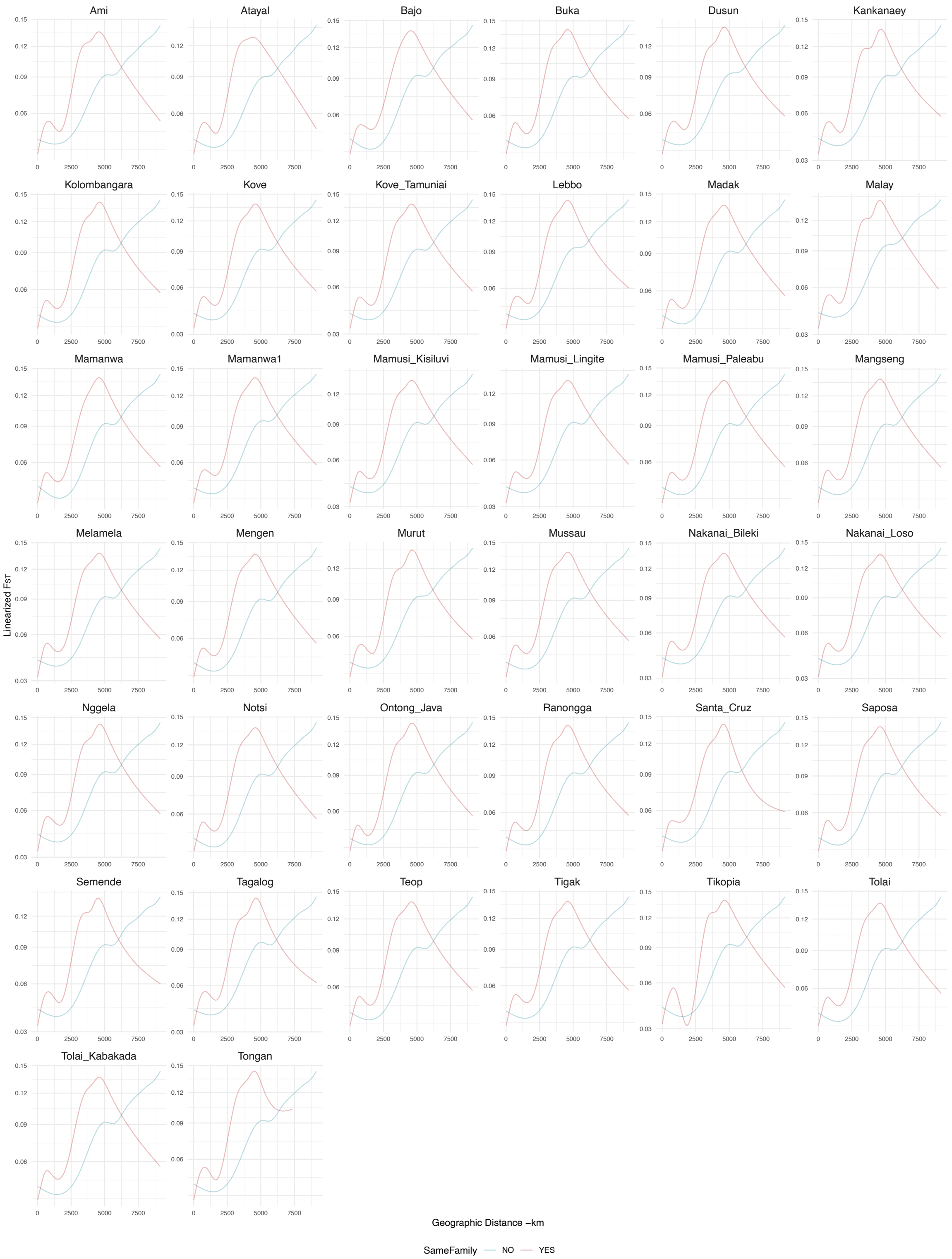

Fig. S19. Correspondence between genetic distances and geographic distances for the Austronesian language family. Smooth (i.e., generalized additive) regressions summarize the between and within family trends. In each panel, the population indicated in the title is removed from the comparisons, to show how each population from the family influences the overall pattern in Figure S12A.

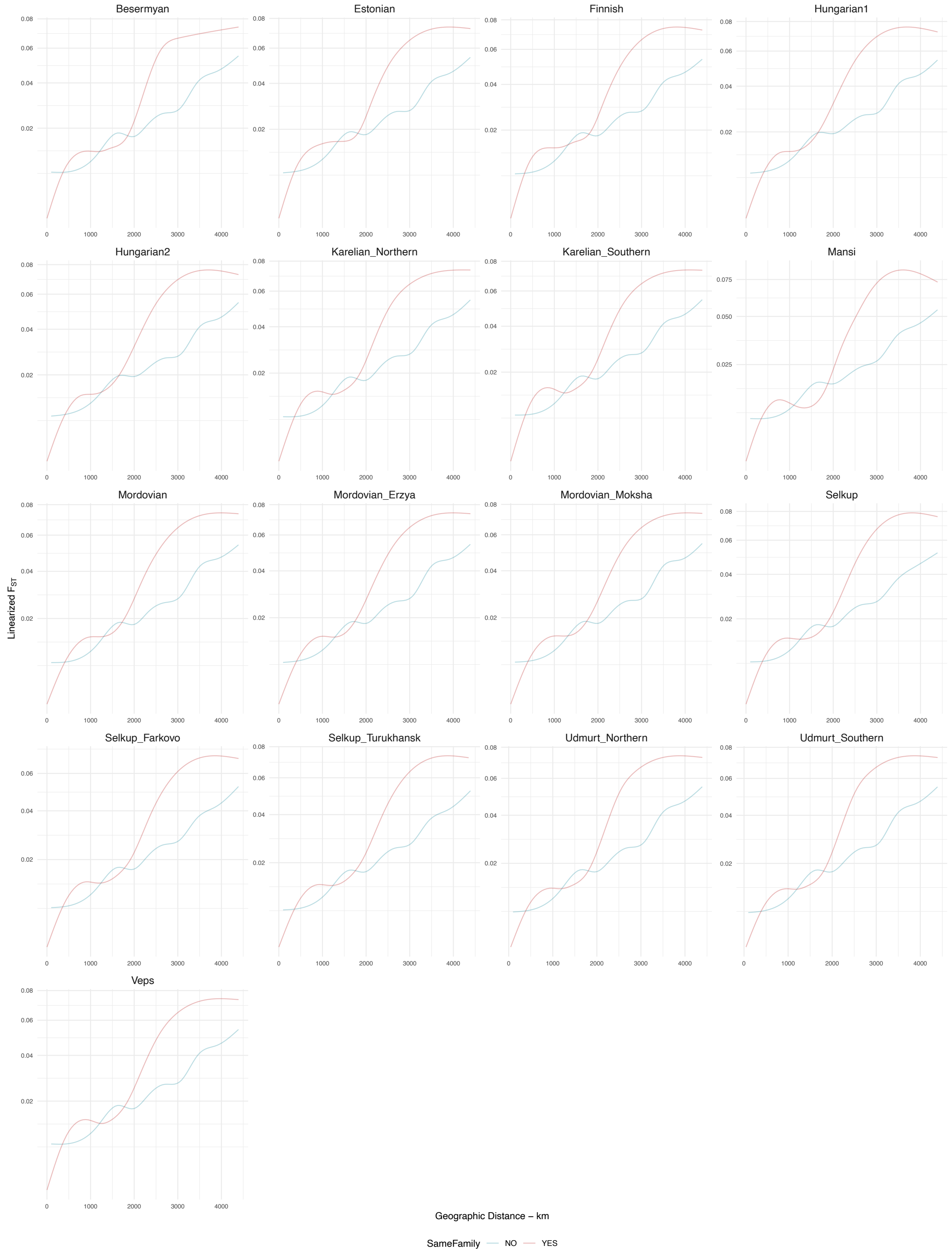

Fig. S20. Correspondence between genetic distances and geographic distances for the Uralic language family. Smooth (i.e., generalized additive) regressions summarize the between and within family trends. In each panel, the population indicated in the title is removed from the comparisons, to show how each population from the family influences the overall pattern in Figure S12A.

3. Genetic and linguistic similarities in the historical timeline

For this analysis we compared the time frame of the genetic divergence against the proposed time frame of language divergence. We only considered pairs of populations that share a most recent common ancestor near the root of the language family. The timing of the genetic divergence distribution can in principle be compared to the proposed divergence time of the proto-language for each language family. It should be noted that not all the pairwise F_{ST} distances can be converted into divergence times, because of the limitations in reconstructing effective population size from Identity By Descent blocks explained in the method section above, however, many can be compared to the linguistic time estimates.

These comparisons are visualized in three figures. First, Figure 3 shows the distribution of genetic divergence times for major language families, excluding drifted populations and marking populations flagged as enclaves and/or misaligned with different symbols. Second, Figure S21A shows these comparisons based on the harmonic mean of the two N_e, which is associated overall with smaller divergence time estimates, to compare the results obtained with a slightly different formula (see Section 1 of the Supplementary). Third, Figure S21B shows the distribution of genetic divergence times for each language family represented by more than one pairwise divergence time, including drifted populations, which can potentially drive deeper divergence times with their large F_{ST} distances.

In Africa, the speakers of Afro-Asiatic languages show a median divergence time of ~3,200 years ago, with some pairs diverging as old as 5,000 and 7,000 years ago. This time frame is more recent than the one suggested by linguistic and archaeological reconstructions, which point towards a very ancient divergence time in the pre-Neolithic (35). The genetic divergence time is more similar to the time range reconstructed with the Generalized Bayesian Dating (GBD) method (36). The Atlantic-Congo language family, here represented by the genetically cohesive Bantu speaking groups, has the bulk of pairwise time divergences compatible with the demographic diffusion associated with a shift to agricultural subsistence starting ~4,000 years ago (37). The reconstructed times are compatible with the harmonic N_e estimates, but the regular mean N_e estimates include pairs that diverged further back in time. For the hunter-gatherer Kx'a, genetic divergence times are found around 2,500 years ago. A linguistic time depth for the family is difficult to reconstruct from historical sources, but the genetic divergence times available are compatible with the GBD results (36). For the neighboring Khoe-Kwadi, a possible origin and migration with pastoralist groups is postulated. The migration is indicated from archaeological data to be at least older than 2,000 years ago (38), and corresponds to some of the genetic divergence dates reconstructed. Younger divergence cases fall within the timing suggested by the GBD methods.

In the Americas, Tupí populations are associated with very ancient divergence times around 7 kya, but the heterogeneous genetic composition and presence of the highly drifted Karitiana and Suruí, together with a relatively small sample size, suggests caution in interpreting the result. The high F_{ST} distances of these populations would be too ancient to be reconciled with the origin and spread of the family. A possible divergence time of ~3-5 kya has been proposed for the Tupí language family expansion, based on glottochronological data (36, 39), and archaeological evidence associated with the agricultural transformation of the landscape and a putative Tupí pottery style (40–42). The Quechua family also presents cases of language shift, showing a genetically cohesive core in the central-southern Andes – where the family might have originated (43) – and where we see matches according to the stringent enclave criteria. This genetically cohesive core is contrasted with the presence of Quechua lowland speakers on the eastern slope of the Andes who have a distinct Amazonian ancestry (10). The overall divergence frame is too ancient to fit the time range from the GBD method and be reconciled with the historical paths that link the diffusion of the Quechua family to the early expansion of the Wari empire starting ~1,400 years ago (44, 45).

In Eurasia, Uralic speakers do not show signals of genetic cohesiveness, as seen in the previous analysis. One of the oldest divergence times for this language family (as reconstructed with quantitative or historical linguistic methods) is of 6-7 kya, (46, 47), while other authors suggest more recent dates of 4-5 kya (48). While a few comparisons could be consistent with the oldest date proposed, the tail of older population divergences in the genetic data confirms that most

comparisons include populations with a very divergent genetic history. This finding suggests that genetically unrelated groups who diverged older than 7 kya ago adopted Uralic languages as a result of cultural exposure without substantial demographic influences. Nevertheless, some pairwise comparisons show much recent divergence times, especially with the harmonic N_e estimates. Recent studies have confirmed this strong geographic substructure for Uralic speakers, but also described a potential, more recent and subtle demographic exchange between long distance Uralic speakers (29), which is not detectable with our F_{ST} analysis. For Nakh-Daghestanian (here represented only by pairs within the Daghestanian subfamily), a very ancient root has been proposed, up to 8 kya (49): this language family presents one rare situation where the genetic divergence times are more recent than the ones reconstructed for language divergence, also more recent than the time frame reconstructed with the GBD method.

In eastern Asia, a relatively shallow historical time around 2 kya has been proposed for the Tungusic family (50, 51). Of our two divergence times reconstructed, excluding one flagged with a misaligned population, one is compatible with this archaeological and historical estimate, and the other one at ca 3 kya is at the extremes of the range reconstructed with the GBD method. The Turkic family is associated with a similarly shallow origin around 2,500-2,000 years ago, based on contact linguistics (50, 52), and supported by Bayesian approaches calibrated with the Seljuk conquest of Baghdad (1055 CE), as the latest date for the divergence of Seljuk-derived languages (Turkish, Azeri, Gagauz) from other Oghuz languages (Turkmen) (53). Only a small number of the divergence time available fits this frame, or the slightly older one reconstructed with the GBD method, while most comparisons (including populations already flagged as misaligned) are much older, and a few even younger than these dates (especially with the harmonic N_e estimates). For Indo-European, we consider the classic dichotomy between the old chronology / Anatolian hypothesis at ~8,000 years ago (54) and the recent chronology / Kurgan steppe hypothesis 5,500 – 6,500 years ago (55). The genetic divergence time seems quite old overall, not fitting with the recent chronology but exceeding the limits of the old chronology as well – while the harmonic N_e estimates could also be included in the recent chronology time frame.

Finally, looking at Southeast Asia and the Pacific, very old dates are reconstructed for the Austronesian family, which includes only populations already flagged as misaligned. For this language family, divergence time has been associated with a population expansion from Taiwan towards the Pacific, starting ~5,500 years ago (56, 57).

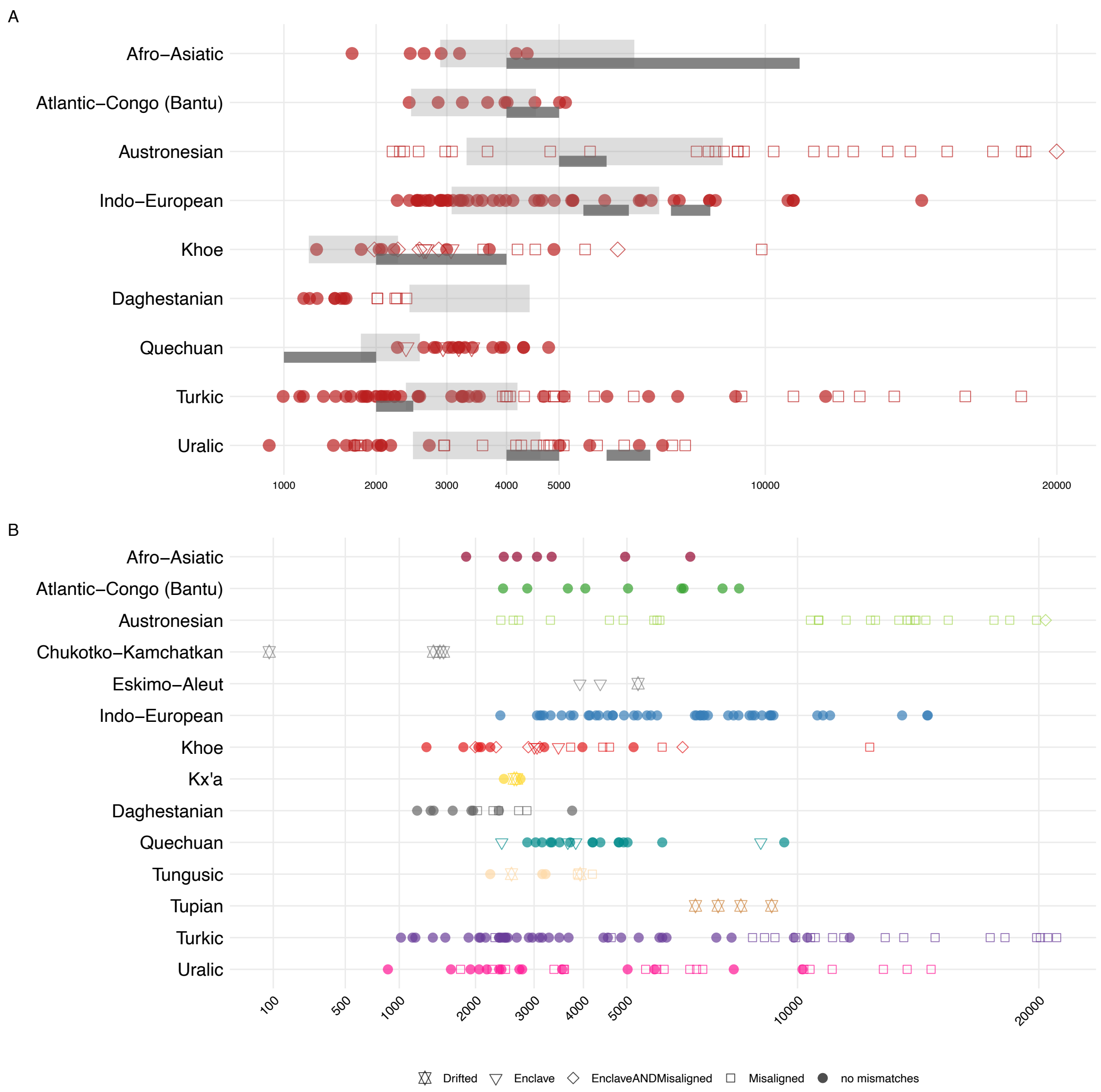

Fig. S21. Pairwise divergence time within families or major subgroups. Each point corresponds to the genetic divergence time of population pairs which share a most recent common ancestor at the root of the language family. Solid circles do not include populations identified as mismatches or drifted. Other symbols indicate pairs which include one population previously flagged as mismatch (enclave and/or misalignment). **A:** Major language families. The genetic divergence times are calculated with the harmonic mean of the two population sizes. Two methods to reconstruct the divergence time of each language family are shown: light gray blocks correspond to the 95% credible intervals of divergence time reconstructed by generalized Bayesian dating (36); darker lines below the gray blocks show proposed divergence times from archaeological and historical reconstructions, with indicative time boundaries. Note that such reconstructions are not available for all language families, and in some cases two historical reconstructions have been suggested for the same family (see Methods and Supplementary Text for references). **B.** All language families with available genetic time divergence reconstruction. Drifted populations are also included in the analysis.

4. Linguistic time divergence distances for single language families

We focus on three language families to explore the gene-language correlation with one measure of linguistic distance: divergence time. Both genetic divergence times and F_{ST} distances are compared against linguistic divergence times for target language families from published sources. Linguistic time splits are extrapolated from trees built with Bayesian statistical methods from lexical dataset based on cognate sets. These reconstructions can be applied only within an established language family, and not across distinct families. External calibration points are used by the authors to anchor the language tree to a time scale, often working with relaxed clock models to allow for the diversification rate to vary across branches. Six linguistic publications have been considered, with the following number of languages matching one or more populations from our genetic database: 32 for Indo-European (data from (58)), 26 for Austronesian (56), 19 for Turkic (53), plus a second Indo-European dataset for 16 matches (59) and second Turkic dataset for 20 matches (60). These last two dataset provided either too recent linguistic splits or a poor coverage when compared to our available genetic divergence times: the results are shown in Figure S22. The reduced number of matches is again due to the fact that not all the genetic populations were usable to calculate N_e and thus the divergence time.

Tree topologies are reconstructed from F_{ST} distances, considering genetic populations as taxa. The corresponding linguistic tree is reconstructed for the same number of populations included in GeLaTo for which there is overlap. For different genetic populations who speak the same language, a linguistic distance of zero is applied. The cognacy based trees from the original linguistic publications are compared against Neighbor-Joining trees from the matrix of F_{ST} distance and plotted in Figure 4 (panels A-C). For visualization purposes, the two trees are displayed one against each other with optimal branch order. The result replicates the classic Cavalli-Sforza display (61) and can be inspected to highlight correspondences and differences.

Quartet analysis is performed to estimate overall similarities between the two tree topologies (Fig. S23). The proportion of identical quartets in the Indo-European trees is 0.68, in the Austronesian trees is 0.65 and in the Turkic is 0.57. The value ranges from 1 for a perfect match between the two trees, to zero when all branches are different.

The Indo-European trees are the ones with the larger number of correspondences. Focusing on the mismatches, the early diverging linguistic position of Greek and Albanian groups is not corresponded in the genetic tree; however, it should be noted that they branch off at a very early position in comparison to the other West Eurasian groups (together with the Sicilians). The two Sicilian populations are therefore not in correspondence with their linguistic relatives, the Italian-speaking populations from Tuscany, Bergamo and Sardinia. German Lipsians are closer to Slavic-speaking Czech instead of their linguistic relatives from the Scandinavian groups. Moldavian and Romanian are genetically closer to neighboring Slavic-speaking groups. Similar patterns have been previously noted in gene-language studies dedicated to West Eurasia (62).

In the Austronesian trees, a similar topology corresponds to the early diverging linguistic stocks in Taiwan, the Philippines and surrounding regions. In Near Oceania, the Kove, Nakanai and Manseng, which are in the same linguistic branch, are not closely related on the genetic side, but are instead connected to various Western Oceanic linkage speaking populations. Polynesian-speaking populations are genetically related.

For the Turkic family, mismatches are numerous. The North Kipchak Tatar and Bashkir do not share preferential genetic connections. A similar mismatch profile is found for the South Kipchak (Kazakhs and Nogai) and the Turkestan Turkic (Uzbek and Uygur). Correspondences between groups that are both linguistically and genetically related are found in the Nuclear Oghuz branch, spoken in Anatolia and in the Caucasus. The linguistic tree available from (53) suggests a linguistic relationship between South Siberian Sayan (Tuvinian and Tofalar) and North Siberian Yakut, which is paralleled by their genetic relatedness.

The correspondence between linguistic and genetic diversity within subbranches of each language family is also applied on a language-based approach (Fig. S24). Here the linguistic divergence time is considered against the genetic divergence time. Each taxon is a language (not a population, like in the previous set of tree comparisons), and divergent values for speakers of the same language are condensed for each node: the maximum and mean value of all the divergence time is taken for multiple populations speaking the same language and for the upstream genetic coalescences. Finally, for each node, the proportion of the mean linguistic and genetic split time is reported. Turkic is the language family for which linguistic and genetic comparisons show the least correspondences. In Indo-European, the main exception is the branch with Baluchi, Kurdish, Persian and Tadzik, for which the genetic reconstructed divergence times are twice more recent than the linguistic divergence times. In Austronesian, maximum genetic divergence times are particularly ancient, due to possible admixture with pre-Austronesian genetic substrate discussed in the main text. Mean genetic divergence times for splits of major subgroups of the language phylogeny are roughly concordant with the linguistic divergence times. Similar parallels are found with the divergence times within the Polynesian linguistic branch and with the split of Bajo. Within the other Austronesian linguistic branches represented in GeLaTo, genetic divergence times are up to three times older than the linguistic divergence times.

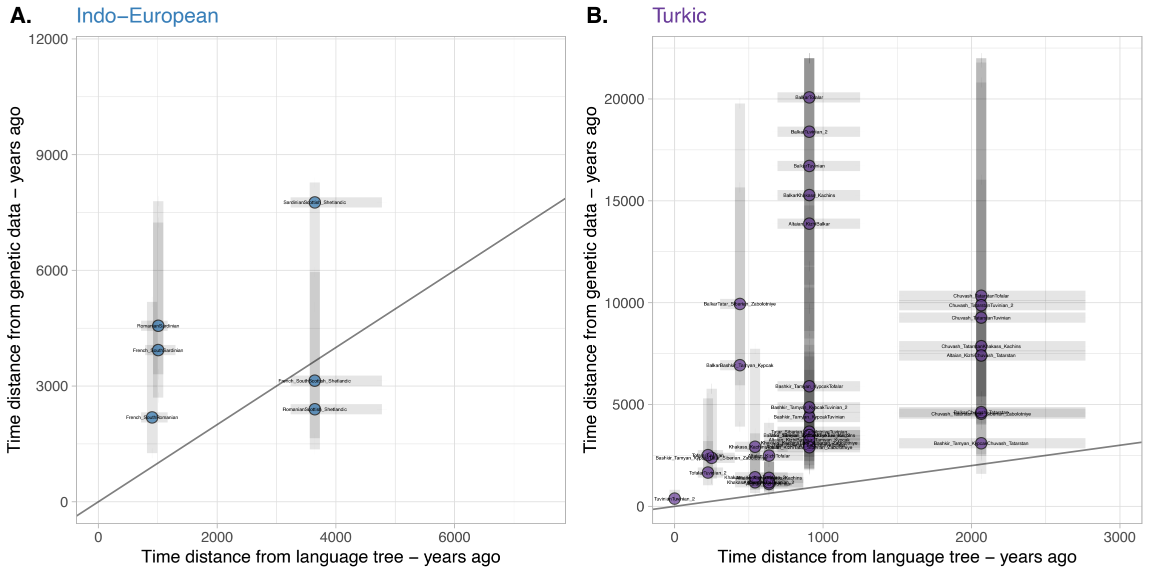

Fig. S22. Correlation between genetic and linguistic divergence times. **A.** Indo-European from Chang et al. 2015; **B.** Turkic from Savelyev & Robbeets, 2020. The black line marks a 1:1 correspondence.

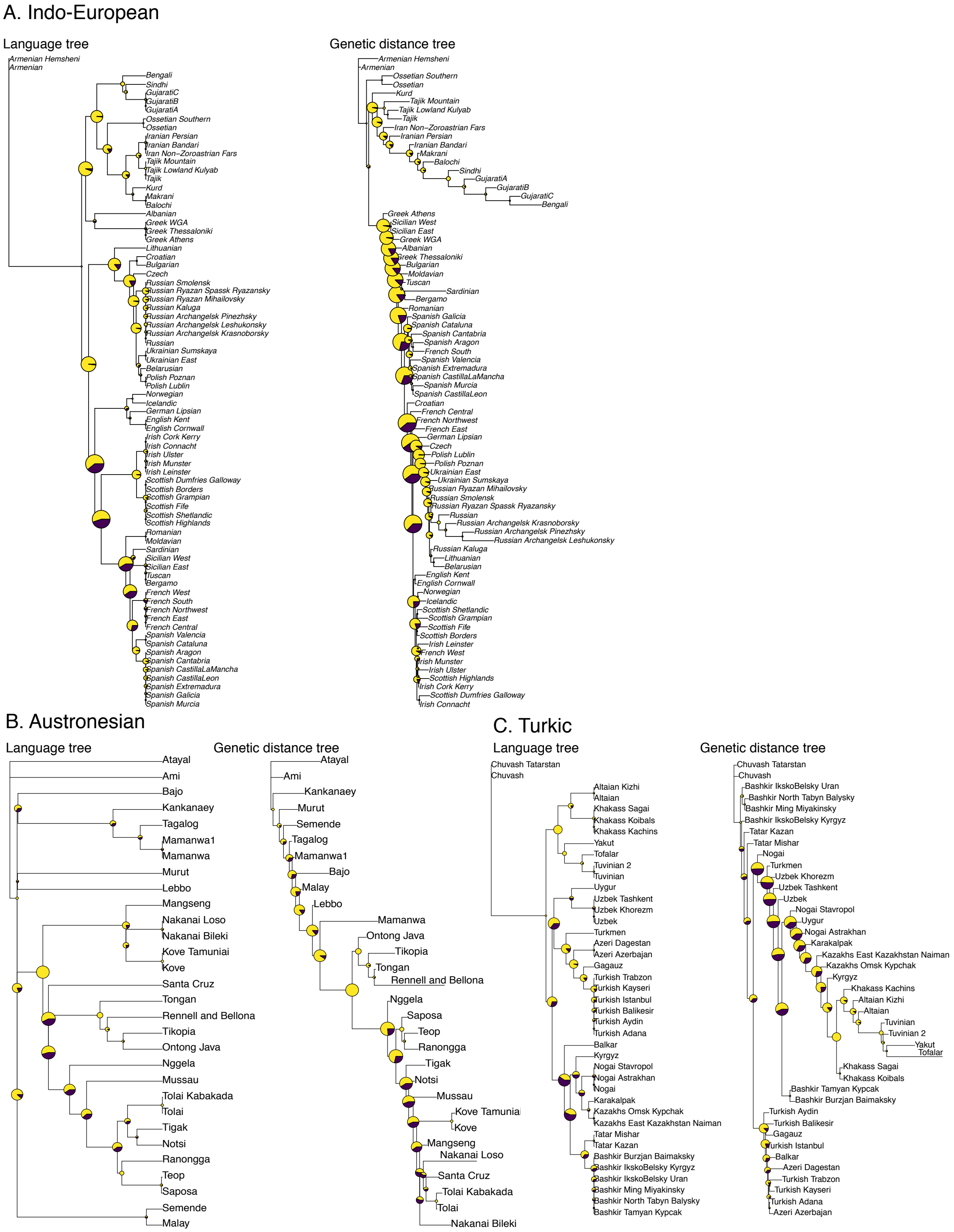

Fig S23. Quartet analysis. Pie chart showing the proportion of quartets in agreement, sized according to the number of quartets influenced by each split. Trees reconstructed from linguistic distances are on the left, trees reconstructed from F_{ST} genetic distances are on the right. **A.** Indo-European. **B.** Austronesian. **C.** Turkic.

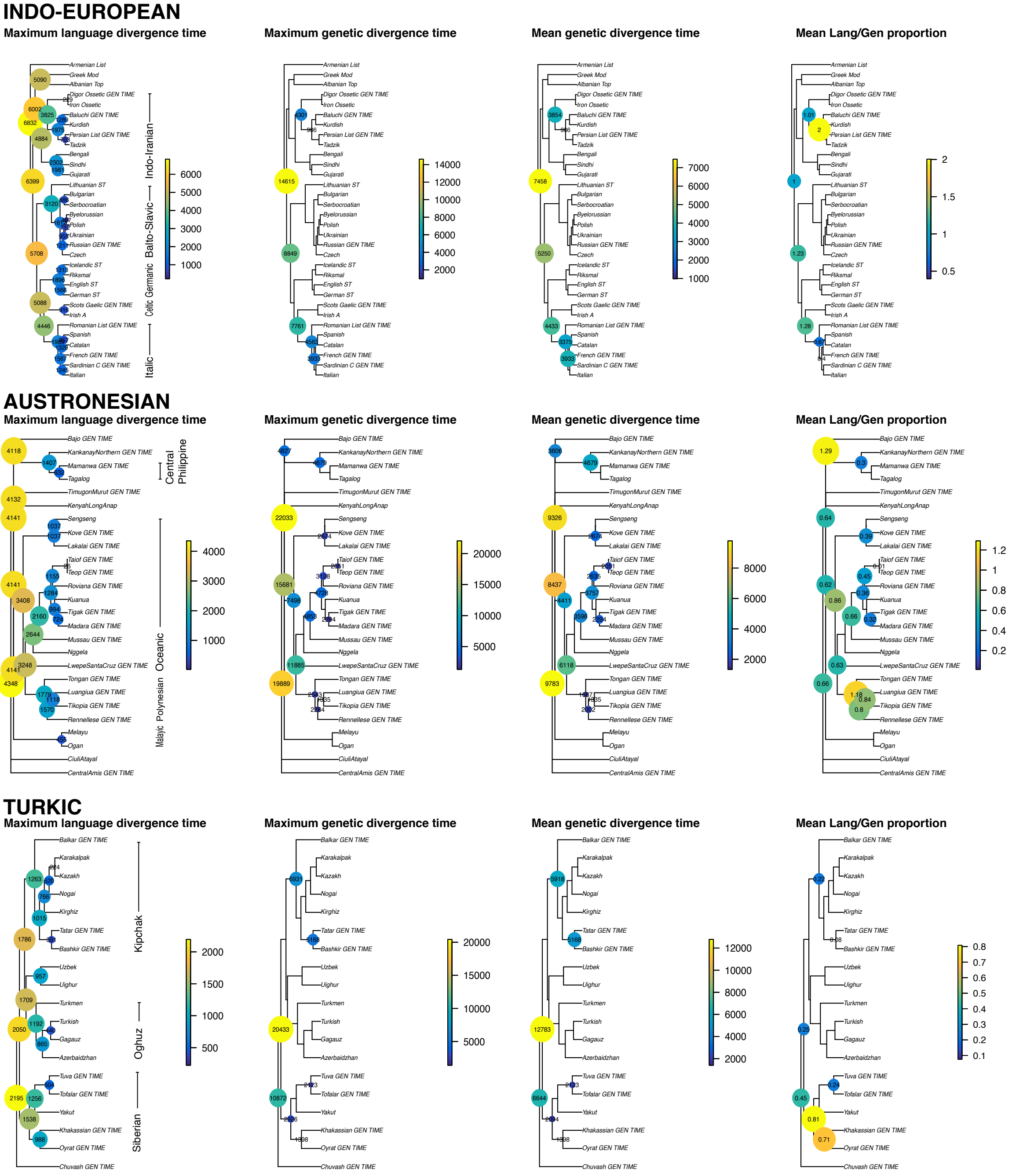

Fig. S24. Language based correspondence of genetic divergence times over linguistic phylogenies. Major language family sub-branches are indicated with vertical text. We retrieved the language divergence time trees from the original publications (with the original language names) and extracted a subset corresponding to populations for which there is a genetic representative in GeLaTo. We plot the genetic divergence times available in GeLaTo (note that not all populations can be used to reconstruct the divergence time) over the linguistic phylogenetic structure. For each pair of languages, the mean of the genetic divergence time was calculated (to account for different genetic populations who speak the same language).

Dataset S1 (separate file). The table includes information on the 397 genetic populations included in the analyses: metadata on language association, geographic location, reference source of the data, sample size, and parameters of genetic relatedness calculated for the analyses.

Dataset S2 (separate file). The table includes information on the 157,212 pairwise comparisons for the populations included in the analyses: F_{ST} genetic distances, geographic distances, genetic divergence times, and divergence times from linguistic publications.

SI References

1. N. Patterson, *et al.*, Ancient admixture in human history. *Genetics* **192**, 1065–1093 (2012).
2. J. K. Pickrell, *et al.*, The genetic prehistory of southern Africa. *Nat Commun* **3**, 1143 (2012).
3. I. Lazaridis, *et al.*, Ancient human genomes suggest three ancestral populations for present-day Europeans. *Nature* **513**, 409–413 (2014).
4. I. Lazaridis, *et al.*, Genomic insights into the origin of farming in the ancient Near East. *Nature* **536**, 419–424 (2016).
5. P. Qin, M. Stoneking, Denisovan Ancestry in East Eurasian and Native American Populations. *Mol Biol Evol* **32**, 2665–2674 (2015).
6. P. Skoglund, *et al.*, Genetic evidence for two founding populations of the Americas. *Nature* **525**, 104–108 (2015).
7. P. Skoglund, *et al.*, Genomic insights into the peopling of the Southwest Pacific. *Nature* **538**, 510–513 (2016).
8. P. Skoglund, *et al.*, Reconstructing prehistoric African population structure. *Cell* **171**, 59–71 (2017).
9. F. Broushaki, *et al.*, Early Neolithic genomes from the eastern Fertile Crescent. *Science (1979)* **353**, 499–503 (2016).
10. C. Barbieri, *et al.*, The current genomic landscape of western South America: Andes, Amazonia and Pacific Coast. *Mol Biol Evol* **36**, 2698–2713 (2019).
11. M. Lipson, *et al.*, Ancient West African foragers in the context of African population history. *Nature* **577**, 665–670 (2020).
12. C. Jeong, *et al.*, The genetic history of admixture across inner Eurasia. *Nat Ecol Evol* **3**, 966–976 (2019).
13. P. Flegontov, *et al.*, Palaeo-Eskimo genetic ancestry and the peopling of Chukotka and North America. *Nature* **570**, 236–240 (2019).
14. H. Hammarström, M. Haspelmath, R. Forkel, Glottolog 4.3 (2020) https:/doi.org/https://doi.org/10.5281/zenodo.4061162.
15. B. S. Weir, C. C. Cockerham, Estimating F-Statistics for the Analysis of Population Structure. *Evolution (N Y)* **38**, 1358–1370 (1984).
16. C. C. Chang, *et al.*, Second-generation PLINK: Rising to the challenge of larger and richer datasets. *Gigascience* **4**, 7 (2015).
17. R Core Team, R: A Language and Environment for Statistical Computing. *R Foundation for Statistical Computing, Vienna, Austria*, http://www.R-project.org/ (2022).
18. M. Nei, *Molecular evolutionary genetics* (Columbia University Press, 1987).
19. G. Bhatia, N. Patterson, S. Sankararaman, A. L. Price, Estimating and interpreting FST: The impact of rare variants. *Genome Res* **23**, 1514–21 (2013).
20. S. R. R. Browning, B. L. L. Browning, Accurate Non-parametric Estimation of Recent Effective Population Size from Segments of Identity by Descent. *Am J Hum Genet* **97**, 404–418 (2015).
21. B. L. Browning, S. R. Browning, Improving the accuracy and efficiency of identity-by-descent detection in population data. *Genetics* **194**, 459–471 (2013).
22. F. Rousset, Genetic differentiation and estimation of gene flow from F-statistics under isolation by distance. *Genetics* **145**, 1219–1228 (1997).
23. P. G. Meirmans, The trouble with isolation by distance. *Mol Ecol* **21**, 2839–2846 (2012).
24. S. Dray, P. Legendre, P. R. Peres-Neto, Spatial modelling: a comprehensive framework for principal coordinate analysis of neighbour matrices (PCNM). *Ecol Modell* **196**, 483–493 (2006).
25. R. Nielsen, *et al.*, Tracing the peopling of the world through genomics. *Nature* **541**, 302–310 (2017).
26. P. Skoglund, D. Reich, A genomic view of the peopling of the Americas. *Curr Opin Genet Dev* **41**, 27–35 (2016).
27. M. Lipson, *et al.*, Population Turnover in Remote Oceania Shortly after Initial Settlement. *Current Biology* **28**, 1157-1165.e7 (2018).

28. C. M. Schlebusch, *et al.*, Southern African ancient genomes estimate modern human divergence to 350,000 to 260,000 years ago. *Science (1979)* **358**, 652–655 (2017).
29. K. Tambets, *et al.*, Genes reveal traces of common recent demographic history for most of the Uralic-speaking populations. *Genome Biol* **19**, 139 (2018).
30. P. Santos, *et al.*, More Rule than Exception: Parallel Evidence of Ancient Migrations in Grammars and Genomes of Finno-Ugric Speakers. *Genes (Basel)* **11**, 1491 (2020).
31. V. J. Moreno-Mayar, *et al.*, Early human dispersals within the Americas. *Science (1979)* **362**, eaav2621 (2018).
32. M. Vicente, M. Jakobsson, P. Ebbesen, C. M. Schlebusch, Genetic Affinities among Southern Africa Hunter-Gatherers and the Impact of Admixing Farmer and Herder Populations. *Mol Biol Evol* **36**, 1849–1861 (2019).
33. A. C. N. Barnard, *Hunters and herders of southern Africa: a comparative ethnography of the Khoisan peoples* (Cambridge University Press, 1992).
34. C. Barbieri, *et al.*, Unraveling the complex maternal history of Southern African Khoisan populations. *Am J Phys Anthropol* **153**, 435–448 (2014).
35. C. Ehret, S. O. Keita, P. Newman, The origins of Afroasiatic. *Science (1979)* **306**, 1680 (2004).
36. T. Rama, S. Wichmann, A test of Generalized Bayesian dating: A new linguistic dating method. *PLoS One* **15**, e0236522 (2020).
37. P. Mitchell, P. Lane, Eds., *The Oxford Handbook of African Archaeology* (Oxford University Press, 2013) https:/doi.org/10.1179/146195714x13820028180603.
38. T. Güldemann, A linguist's view: Khoe-Kwadi speakers as the earliest food-producers of southern Africa. *Southern African Humanities* **20**, 93–132 (2008).
39. R. S. Walker, S. Wichmann, T. Mailund, C. J. Atkisson, Cultural Phylogenetics of the Tupi Language Family in Lowland South America. *PLoS One* **7**, e35025 (2012).
40. G. Urban, On the geographical origins and dispersion of Tupian Languages. *Revista de Antropologia* **39**, 61–104 (1996).
41. A. Rodrigues, A. Cabral, "Tupian" in *The Indigenous Languages of South America.*, L. Campbell, V. Grondona, Eds. (Mouton de Gruyter, 2012), pp. 495–574.
42. F. S. Noelli, The Tupi: Explaining origin and expansions in terms of archaeology and of historical linguistics. *Antiquity* **72**, 648–663 (1998).
43. W. F. H. Adelaar, Modeling convergence: Towards a reconstruction of the history of Quechuan-Aymaran interaction. *Lingua* **122**, 461–469 (2012).
44. D. Beresford-Jones, P. Heggarty, "Andes: archaeology" in *The Encyclopedia of Global Human Migration*, I. Ness, P. Bellwood, Eds. (Blackwell Publishing Ltd, 2013), pp. 410–16.
45. W. H. Isbell, La arqueología wari y la dispersión del quechua. *Boletín de Arqueología PUCP*, 199–220 (2010).
46. T. Honkola, *et al.*, Cultural and climatic changes shape the evolutionary history of the Uralic languages. *J Evol Biol* **26**, 1244–1253 (2013).
47. P. Sammallahti, "Historical phonology of the Uralic languages" in *The Uralic Languages: Description, History and Foreign Influences*, D. Sinor, Ed. (Brill, 1988), pp. 478–554.
48. J. Janhunen, Proto-Uralic–what, where, and when. *The quasquicentennial of the Finno-Ugrian society* **258**, 57-78. (2008).
49. J. Nichols, "The vertical archipelago: Adding the third dimension to linguistic geography" in *Space in Language and Linguistics*, P. Auer, M. Hilpert, A. Stukenbrock, B. Szmrecsanyi, Eds. (De Gruyter, 2013) https:/doi.org/10.1515/9783110312027.38.
50. M. Robbeets, R. Bouckaert, Bayesian phylolinguistics reveals the internal structure of the Transeurasian family. *J Lang Evol* **3**, 145–162 (2018).
51. J. Janhunen, The expansion of Tungusic as an ethnic and linguistic process. *Recent advances in Tungusic linguistics*, 5–16 (2012).
52. J. Janhuen, "Reconstructing the Language Map of Prehistorical Northeast Asia" in *Anantam Śāstram: Indological and Linguistic Studies in Honour of Bertil Tikkanen*, K. Karttunen, Ed. (Suomalais Ugrilainen Seura, 2009), pp. 283 –305.
53. D. J. Hruschka, *et al.*, Detecting Regular Sound Changes in Linguistics as Events of Concerted Evolution. *Current Biology* **25**, 1–9 (2015).

54. C. Renfrew, Archaeology and language: the puzzle of Indo-European origins. *Curr Anthropol* **29**, 437–468 (1990).
55. D. W. Anthony, D. Ringe, The Indo-European Homeland from Linguistic and Archaeological Perspectives. *Annu Rev Linguist* **1**, 199–219 (2015).
56. R. D. Gray, A. J. Drummond, S. J. Greenhill, Language phylogenies reveal expansion pulses and pauses in pacific settlement. *Science (1979)* **323**, 479–483 (2009).
57. R. A. Blust, *The Austronesian Languages* (Pacific Linguistics, Research School of Pacific and Asian Studies, Australian National University, 2009).
58. R. Bouckaert, *et al.*, Mapping the origins and expansion of the Indo-European language family. *Science (1979)* **337**, 957–960 (2012).
59. W. Chang, *et al.*, Ancestry-constrained phylogenetic analysis supports the Indo-European steppe hypothesis. *Language (Baltim)* **91**, 194–244 (2015).
60. A. Savelyev, M. Robbeets, Bayesian phylolinguistics infers the internal structure and the time-depth of the Turkic language family. *J Lang Evol* **5**, 39–53 (2020).
61. L. L. Cavalli-Sforza, Genes, peoples and languages. *Sci Am* **265**, 104–110 (1991).
62. G. Longobardi, *et al.*, Across language families: Genome diversity mirrors linguistic variation within Europe. *Am J Phys Anthropol* **157**, 630–640 (2015).
